# p63, a key regulator of Ago2, links to the microRNA-144 cluster

**DOI:** 10.1038/s41419-022-04854-1

**Published:** 2022-04-22

**Authors:** Benfan Wang, H. Helena Wu, Yasser Abuetabh, Sarah Leng, Sandra T. Davidge, Elsa R. Flores, David D. Eisenstat, Roger Leng

**Affiliations:** 1 Department of Laboratory Medicine and Pathology, 370 Heritage Medical Research Center, University of Alberta, Edmonton, AB T6G 2S2 Canada; 2 Department of Obstetrics & Gynecology & Physiology, 232 Heritage Medical Research Center, University of Alberta, Edmonton, AB T6G 2S2 Canada; 3 Department of Molecular Oncology, H. Lee Moffitt Cancer Center, 12902 Magnolia Drive, Tampa, FL 33612 USA; 4 Department of Oncology, Cross Cancer Institute, 11560 University Ave., University of Alberta, Edmonton, AB T6G 1Z2 Canada; 5 Department of Pediatrics, University of Alberta, 11405 - 87 Ave., Edmonton, AB T6G 1C9 Canada; 6 Murdoch Children’s Research Institute, Department of Paediatrics, University of Melbourne, 50 Flemington Road, Parkville, VIC 3052 Australia

**Keywords:** Cancer, Molecular biology

## Abstract

**Abstract:**

As a key component of the RNA-induced silencing complex (RISC), Argonaute2 (Ago2) exhibits a dual function regulatory role in tumor progression. However, the mechanistic basis of differential regulation remains elusive. p63 is a homolog of the tumor suppressor p53. p63 isoforms play a critical role in tumorigenesis and metastasis. Herein, we show that p63 isoforms physically interact with and stabilize Ago2. Expression of p63 isoforms increases the levels of Ago2 protein, while depletion of p63 isoforms by shRNA decreases Ago2 protein levels. p63 strongly guides Ago2 dual functions in vitro and in vivo. Ectopic expression of the miR-144/451 cluster increases p63 protein levels; TAp63 transactivates the miR-144/451 cluster, forming a positive feedback loop. Notably, miR-144 activates p63 by directly targeting Itch, an E3 ligase of p63. Ectopic expression of miR-144 induces apoptosis in H1299 cells. miR-144 enhances TAp63 tumor suppressor function and inhibits cell invasion. Our findings uncover a novel function of p63 linking the miRNA-144 cluster and the Ago2 pathway.

**Facts and questions:**

- Identification of Ago2 as a p63 target.
- Ago2 exhibits a dual function regulatory role in tumor progression; however, the molecular mechanism of Ago2 regulation remains unknown.
- p63 strongly guides Ago2 dual functions in vitro and in vivo.
- Unraveling a novel function of p63 links the miRNA-144 cluster and the Ago2 pathway.

## Introduction

As essential components of the RNA-induced silencing complex (RISC), the Argonaute protein family plays a central role in RNA silencing processes [1–5]. The Argonaute2 (Ago2) protein, encoded by the *E1F2C2* gene, provides a catalytic engine for RNA interference (RNAi) [6, 7]. Ago2 is essential for murine embryonic development since knockout of the *Ago2* gene is lethal [6, 8]. Ago2 exhibits a dual function regulatory role in tumor progression. Overexpression of Ago2 accelerates malignant transformation in some tumors and is associated with low overall survival of some cancer patients [2, 9–11]. However, Ago2 expression is downregulated in melanoma, primary lung cancer, and invasive breast carcinoma [12–14]. The mechanisms underlying these two opposite functions of Ago2, which promote or inhibit tumor growth, remain unknown.

p63 is a homolog of the tumor suppressor p53. p63 has two different promoters, a 5ʹ promoter (P1) that precedes the first exon encoding the full-length p63 protein designated TAp63 and an alternative promoter (P2) that is a cryptic 3ʹ intronic promoter encoding an N-terminal truncated variant of p63 named ΔNp63 [15–17]. TAp63 transactivates many p53 target genes, leading to apoptosis and cell cycle arrest [18–20]. ΔNp63 blocks the function of p53 and TAp63 in a dominant-negative manner [16–18]. p63 is rarely mutated in human tumors [21, 22]. p63 deficiency is embryonic lethal in mice, causing severe developmental abnormalities [23, 24]. More aggressive, metastatic tumors lose TAp63 expression, suggesting that loss of TAp63 accelerates tumorigenesis and metastatic spread [25–27].

miRNAs regulate the expression and function of more than 30% of all genes, and recent studies have further revealed the importance of miRNAs and miRNA processing in tumorigenesis [28–30]. The miR-144/451 cluster is highly conserved in different species [31, 32]. The miR-144/451 locus is required for erythroid homeostasis [31, 32]. The levels of miR-144/451 are downregulated in a large number of human tumors, including breast carcinoma and non-small-cell lung carcinoma (NSCLC) [33–38]. Only a few studies have reported that miR-144 promotes cell proliferation [39]. The premiR-451 hairpin is the only hairpin directly cleaved by Ago2 to generate mature miR-451 [40, 41]. However, the mechanism by which miR-144 regulates miR-451 processing by Ago2 remains unknown.

We show that p63 is a crucial regulator of Ago2 that functions as a tumor suppressor or as an oncogene. We present evidence that p63 binds and transcriptionally activates Ago2. p63 isoforms modulate Ago2 function in tumor progression. We found that the expression of miR-144/451 increased p63 protein levels, and TAp63 transactivated the promoter of the miR-144/451 cluster, forming a positive feedback loop. Furthermore, miR-144 induces apoptosis and suppresses cell invasion in a TAp63-dependent manner.

## Material and methods

### Cell culture and DNA transfection

All cells except for the indicated cells were maintained in α-Minimal Essential Medium (α-MEM) supplemented with 10% fetal bovine serum. MRC-5 and H1792 cells were maintained in Dulbecco’s modified Eagle’s medium (DMEM) supplemented with 10% fetal bovine serum. Wild-type MEFs, TAp63^−/−^ MEFs, and ΔNp63^−/−^ MEFs were provided by Dr. E. Flores [42] and cultured in Dulbecco’s modified Eagle’s medium (DMEM, Gibco, Invitrogen) containing 10% FBS (Gibco, Invitrogen). Cells were transfected using the calcium phosphate method or Lipofectamine 2000 (Invitrogen).

### Plasmids and antibodies

p21-Luc, pCMV-Bam-MDM2, and His-Ubiquitin (wt) have been described previously. myc-AIP4 was obtained from Dr. Tony Pawson (University of Toronto), and His-tagged ubiquitin (Ub) was provided by Dr. Wei Gu (Columbia University, USA). Ago2 was cloned into p3×Flag- CMV-10 (Sigma). miRNA expression vectors were constructed using the pCMV-MIR vector (Origene). All PCR products were confirmed by sequencing. Anti-p63 (DeltaNp63 (E6Q3O), Cell Signaling; Poly 938102 (anti-TAp63), BioLegend), anti-Ago2 (C34C6, Cell Signaling Technology), anti-Flag (M2, M3165, and M5, M4042, Sigma), anti-MDM2 (2A10, OP115, Calbiochem; SMP14, sc-965, Santa Cruz Biotechnology), anti-ubiquitin (550944, BD Bioscience), anti-Itch (ab109018, Abcam), anti-HA (sc-57592, Santa Cruz Biotechnology), anti-GFP (sc-390394, Santa Cruz Biotechnology), anti-p53 (Pab1801, sc-98, DO1, sc-126, Santa Cruz Biotechnology), and anti-actin (612657, BD Bioscience) were used according to the manufacturers’ instructions.

### shRNA experiments

p63 and Ago2 shRNA constructs were cloned into the vector pSUPER-gfp-neo (Oligoengine, USA) using the Bg1II and XhoI restriction sites and confirmed by sequencing. For shRNA experiments, H1299 or SCC9 cells were transfected with the indicated shRNA constructs by using Lipofectamine 2000 (Invitrogen). Cells were placed under G418 selection two days after transfection, and drug-resistant colonies were selected two weeks later. We used the following sequences for these experiments: TAp63-shRNA: GCACACAGACAAATGAATT [43]. ΔNp63-shRNA: CAATGCCCAGACTCAATTT [43]. Ago2 shRNA1: GCAGGACAAAGATGTATTA. Ago2 shRNA2: GCACGGAAGTCCATCTGAA. For the miR-144-3p decoy, double strands of oligonucleotides (AGTACATCATCTATACTGTA) containing six repeated sequences that were completely complementary to miR-144-3p were synthesized (Life Technologies) and inserted into the pMIR-REPORTTM luciferase reporter described previously [44, 45]. For the miR-451 decoy construct, double strands of oligonucleotides (AACTCAGTAATGGTAACGGTTT) containing six repeated sequences that were completely complementary to miR-451 were synthesized and inserted into the pMIR-REPORTTM luciferase reporter [44, 45].

### Immunoprecipitation

Cells were lysed in 50 mM Tris-HCl (pH 8.0), 5 mM EDTA, 150 mM NaCl, and 0.5% NP-40 containing a protease inhibitor tablet (Roche) and immunoprecipitated with specific antibodies. The immune complexes were collected with protein A agarose beads and washed four times with lysis buffer. The immunoprecipitates were analyzed by SDS PAGE, followed by autoradiography.

### Colony formation assay

The clonogenic assay was performed according to previously published protocols [46] with modification. Briefly, 24 h after seeding, H1299 cells were transfected with the indicated expression plasmids. After an additional 24 h, cells were counted and seeded in a new 3.5 cm plate at a density of 1000 cells/well with G418 and incubated for two weeks at 37 °C in a humidified atmosphere of 5% CO2. The colonies were fixed with 4% paraformaldehyde for 10 min, stained with 0.1% crystal violet for 20 min, washed three times with running water, dried at 37 °C, and photographed. The colony numbers were counted.

### Measurement of Ago2 half-life

To measure the Ago2 half-life, H1299 and SCC9 cells were transfected with the indicated plasmid, followed by treatment with 25 μg/ml cycloheximide (CHX) to inhibit de novo protein synthesis. Protein levels were monitored by immunoblotting with an Ago2-specific antibody at the indicated time points.

### Tube formation assay

The HUVEC tube formation assay was carried out as previously described [11]. Trypsinized HUVECs (1 × 10^4^) were seeded onto a Matrigel-coated (Corning, USA) 96-well plate and incubated with cell-free culture supernatants generated from p63 knockdown alone or with Ago2 knockdown or p63 or Ago2 overexpression in H1299 or SCC-9 cells and controls at 37 °C and 5% CO2 for 16 h. The degree of tube formation was evaluated using an inverted microscope. Quantitative analysis was performed using the ImageJ plugin “Angiogenesis Analyzer”.

### Chick CAM assay

The chick embryo metastasis assay was performed [46, 47]. Briefly, H1299 clones (5 × 10^6^ cells) or SCC9 clones (2 × 10^7^ cells) expressing the indicated shRNAs for p63 and/or Ago2 were suspended in Matrigel and then inoculated onto the chick chorioallantoic membrane (CAM) on a developmental day [10]. After 11 days, on developmental day 20, embryos were sacrificed, and primary tumors were removed and imaged at 2X by a dissecting microscope. The typical vascular area (size: 1.5 × 1 inches) was intercepted from the original image (7.2 × 5.6 inches). Neovascularization was quantified by counting the number of capillaries of the typical vascular area (1.5 × 1 inches) on the tumor.

### Cell cycle analysis

The method was described previously [44, 48, 49]. Briefly, H1299 cells were transfected with the indicated miRNAs. After 40 h, the cells were washed, fixed with 70% ethanol, treated with 100 μg/mL RNase A, labeled with 50 μg/mL propidium iodide (PI) for 3 h at 4 °C, and analyzed by flow cytometry (Becton Dickinson). The data were analyzed using FlowJo software (TreeStar Inc).

### Apoptosis assay

As described previously [44, 48, 49], the cells were transfected with the indicated expression plasmids for Annexin V staining (BD, Biosciences). The cells were then trypsinized, washed, and resuspended in PBS containing 25 μg/ml Annexin-V-FITC and 50 μg/ml 7-AAD (7-amino-actinomycin D) prior to FACS analysis. The data were analyzed using FlowJo software (TreeStar Inc).

### Matrigel matrix-coated invasion assay

The effect of miR-144 on the invasive capability of the neoplastic cells was measured by Transwell assay as described previously [44, 48]. For the invasion assay, 2 × 10^4^ cells were seeded into the insert with precoated Matrigel matrix (BD, Biosciences) in 24-well Transwell units. After a 24 h incubation period, the inside of each insert was swabbed with cotton swabs and stained. The cells that had passed through the filter into bottom wells were fixed in methanol for 20 min, stained with 0.1% crystal violet for 15 min, photographed with a photomicroscope (Nikon), quantified by counting using the ImageJ plugin ‘cell counter’ and expressed as a percentage of the control group, with control values set at 100%.

### Luciferase assay

The method was as described previously [44, 48, 49]. Briefly, a double-stranded oligonucleotide containing one copy of putative p53RE was inserted into the luciferase reporter vector pGL3-basic (Promega). We examined three putative p53 binding sites of Ago2 with a lower mismatch rate. H1299 cells were transfected with the indicated expression plasmids. Forty hours later, the cells were lysed with Reporter Lysis Buffer (Promega). Luciferase activity was measured on samples containing equivalent amounts of protein using an LB9507 luminometer and the luciferase assay reagent (Promega); values were normalized to β-galactosidase activity.

### RNA extraction, RT–PCR, and quantitative real-time PCR

The method was described previously [44, 48]. Briefly, total RNA was extracted with TRIzol (Invitrogen). Real-time PCR first-strand cDNA was synthesized from 2 µg of total RNA using SuperScript (Invitrogen). Real-time PCR was performed using the StepOnePlus Real-Time PCR System with SYBR green PCR master mix (Applied Biosystems). The qPCR primers for TAp63,ΔNp63, and GAPDH were described previously (human TA p63 forward: GGACTGTATCCGCATGCAG; human TAp63 reverse: GAGCTGGGCTGTGCGTAG; human ΔNp63 forward: GAAGAAAGGACAGCAGCATTGA; human ΔNp63 reverse: GGGACTGGTGGACGAGGAG; human GAPDH forward: AGCCACATCGCTCAGACAC; human GAPDH reverse: GCCCAATACGACCAAATCC) [26]. Assays to quantify mature miR-144/451 and Itch mRNA levels were performed using the TaqMan^®^ microRNA assay and quantitative real-time PCR (qRT–PCR, Applied Biosystems). The expression levels of miRNA-144, miR-451, and Itch mRNA were normalized to those of U6 snRNA and GAPDH, respectively, by using the comparative Ct method as previously described [44, 45].

### Chromatin immunoprecipitation

The method was described previously [44, 48]. Briefly, cells (3 × 10^7^) were fixed with 1% formaldehyde and neutralized by the addition of 125 mM glycine for 5 min. Cells were washed twice with ice-cold PBS and sonicated to an average fragment size of 500 bp. A 100-μl aliquot of sonicated chromatin (3 × 10^6^ cell equivalents) was used for immunoprecipitation with the indicated antibodies. After overnight incubation at 4 °C, immune complexes were collected and washed sequentially for 10 min once in TSEI (0.1% SDS, 1% Triton X-100, 2 mM EDTA, 20 mM Tris-HCl [pH 8], and 150 mM NaCl), 4 times in TSEII (0.1% SDS, 1% Triton X-100, 2 mM EDTA, 20 mM Tris-HCl [pH 8], 500 mM NaCl), once in buffer III (0.25 M LiCl, 1% NP-40, 1% deoxycholate, 1 mM EDTA, and 10 mM Tris-HCl [pH 8]) and three times in TE. Samples were extracted twice in 250 μl of 1% SDS and 0.1 M NaHCO_3_ and heated at 65 °C overnight to reverse crosslinks. DNA was purified by extraction with phenol–chloroform, precipitated with ethanol, and resuspended in 50 μl TE. A 1 μl aliquot was used in the qRT–PCR. Primers for amplification were as follows: Ago2 promoter 3: 5ʹ-CCCTCCTTCCTCTTTGTAG-3ʹ and 5ʹ- CAGGCAATGGAATAAATCA -3ʹ. Ago2 promoter negative region: 5ʹ CCCCTGACTGCTGGAATGG 3ʹ and 5ʹ- CGGAGCTTGAACTTGAACG -3ʹ; MDM2 intron 1: 5ʹ-GCAGGTTGACTCAGCTTTT-3ʹ and 5ʹ-CACAGGTCTACCCTCCAAT-3ʹ. miR-144/451 promoter (–3548): 5ʹ-GAGGGCAGGTGCCTGTAT-3ʹ and 5ʹ-CGGGTTTCCCAACCTGTAG-3ʹ. miR-144/451 promoter (–1247): 5’- CCCCCTAGTAGGATATGGACTTTT-3ʹ and 5ʹ-CTACTTGGGAGGCTGAGGTG-3ʹ. The PCR cycles were 94 °C for 30 s, 55 °C for 20 s, and 72 °C for 30 s, which were repeated 30 times.

### Ago2 RNA immunoprecipitation (RIP)

Ago2 RNA immunoprecipitation (RIP) was performed [50]. Briefly, cells were lysed in NP40 lysis buffer containing a protease inhibitor tablet (Roche) and inhibitors of RNA. The lysate was immunoprecipitated with Ago2-specific antibodies or with mouse IgG control antibodies. The immunoprecipitated complexes were collected with Sepharose beads, washed four times with lysis buffer, incubated with RNase-free DNase I buffer, and subjected to protease K digestion. Coimmunoprecipitated RNAs were isolated and subjected to qRT–PCR analysis to detect miR-144 and miR-451 expression. Data are presented as means ± Standard Deviation (SD).

### Statistical analysis

Statistical significance was analyzed by a two-tailed Student’s *t*-test and expressed as a *p* value using GraphPad Prism8 software. A *p* value <0.05 was considered significant.

## Results

### Identification of Ago2 as a p63 target

**Fig. 1:**
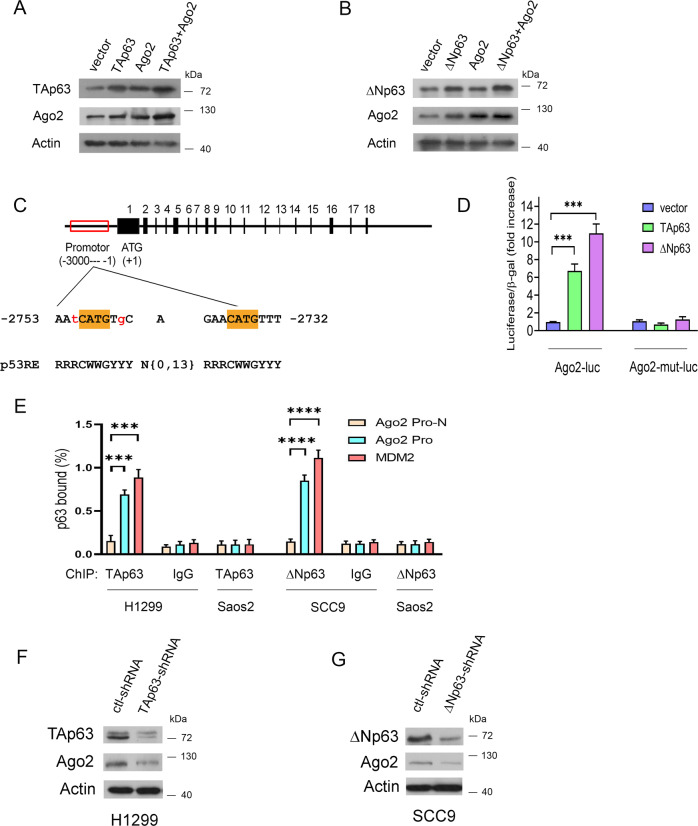
Identification of Ago2 as a p63 target. **A**, **B** H1299 (**A**) or SCC9 cells (**B**) were transfected with TAp63 or ΔNp63 expression plasmids, or in combination with Ago2. The levels of endogenous Ago2 were analyzed by Western blotting. An antibody against β-actin was used as a loading control. **C** The potential p53 binding site in the promoter of the human Ago2 gene is indicated above the p53 consensus binding site, with R denoting purine, Y denoting pyrimidine, and W denoting A or T. The numbering is relative to the first nucleotide of the proposed ATG initiator methionine. The potential p53 binding site in Ago2 matches the consensus at 18 out of 20 nucleotides. **D** Histogram representing the ability of TAp63 or ΔNp63 to transcriptionally activate a luciferase reporter bearing the p53 binding sequence or the corresponding mutation sequence from Ago2 (Ago2-Luc) or Ago2-mut-Luc (containing mutant p53 binding sites). A β-galactosidase reporter construct was included in all the transfection mixes and used for normalization. Error bars indicate SEM (*n* = 5). ***p < 0.001. **E** Chromatin immunoprecipitation assay of p53 DNA binding activity in Saos2, H1299 cells, and SCC9 cells. ChIP-enriched DNA was quantified by qRT–PCR with the indicated specific primers. TAp63-specific (poly938102) and ΔNp63-specific (E6Q30) antibodies or control IgG were used. qRT–PCR analysis (using primers for the promoter of the Ago2 or Ago2 promoter negative region or intron 1 of MDM2) is shown using input DNA (1/20 of ChIP) or DNA after ChIP. The amplification products ranged in length from 150 bp to 200 bp. All experiments were performed in triplicate. ***P < 0.001. ****P < 0.0001. **F**, **G** H1299 (**F**) or SCC9 (**G**) cells were transfected with the indicated shRNA constructs. The amounts of endogenous TAp63 (or ΔNp63) and Ago2 proteins were determined by Western blotting with TAp63 specific- (**F**), ΔNp63 specific- (**G**), and Ago2-specific antibodies.

p63 has been reported to encode sequence-specific nuclear transcription factors that bind to the p53 responsive element (RE) in their target genes [17, 51]. Ago2 was shown to interact with the p53 tumor suppressor [50]. To explore the relationship between p63 and Ago2, we studied tumor cell lines derived from head and neck squamous cell carcinoma (HNSCC) and NSCLC. BJ (normal human fibroblast cell line, ATCC) and MRC-5 (derived from normal lung tissue, ATCC) cells were considered controls for SCC9 and NSCLC, respectively. Ago2 and ΔNp63 were highly expressed in SCC9 cells compared to BJ cells, while Ago2 expression was relatively low in NSCLC cells compared to MRC-5 cells (Supplementary Fig. 1A). qRT–PCR was used to visualize TAp63 and ΔNp63 mRNA expression (Supplementary Fig. 1B). These data suggested that TAp63 is the primary p63 isoform in H1299 cells, while ΔNp63 is the main isoform in SCC9 cells. Online microarray data showed that TAp63 upregulated the expression of Ago2 (Supplementary Fig. 1C). To exclude the vital regulatory role of p53, we selected H1299 and SCC9 cells (expressing no p53 protein, [48, 52, 53]). Ectopic expression of TAp63 or ΔNp63 increased endogenous Ago2 protein levels in H1299 (Fig. 1A) and SCC9 cells (Fig. 1B), respectively, suggesting a role for p63 isoforms in Ago2 regulation. Several putative p53 binding sites were found within the promoter and intron of Ago2 (Supplementary Table 1, p53FamTaG). Saos2 cells (p53-null, no p63 expression [54]) were transfected with the three putative Ago2-Luc constructs, along with plasmids expressing p63 isoforms or an empty vector. TAp63 and ΔNp63 enhanced luciferase reporter gene expression only to the binding site located between –2753 and –2732 of the Ago2 promoter (Fig. 1C). TAp63 moderately enhanced Ago2-Luc luciferase expression by 6.7-fold; in contrast, ΔNp63 increased Ago2-Luc activity by 10.9-fold. Mutation of the p53 binding site had no activity in the assays (Fig. 1D). We then examined the binding ability of p63 to the Ago2 promoter by chromatin immunoprecipitation (ChIP), analyzed by qRT−PCR. Amplification of a region that does not contain p53RE (named Ago2 Pr-N) was used as a negative control, and amplification of a region containing p53RE in the first intron of the MDM2 gene [55] was used as a positive control. Our findings revealed that the Ago2 promoter was bound by p63 in cells (Fig. 1E). Furthermore, depletion of TAp63 (H1299) or ΔNp63 (SCC9) downregulated the Ago2 protein (Fig. 1F, G). Together, these data indicated that p63 isoforms could regulate Ago2 protein levels.

### p63 interacts with and stabilizes Ago2

**Fig. 2:**
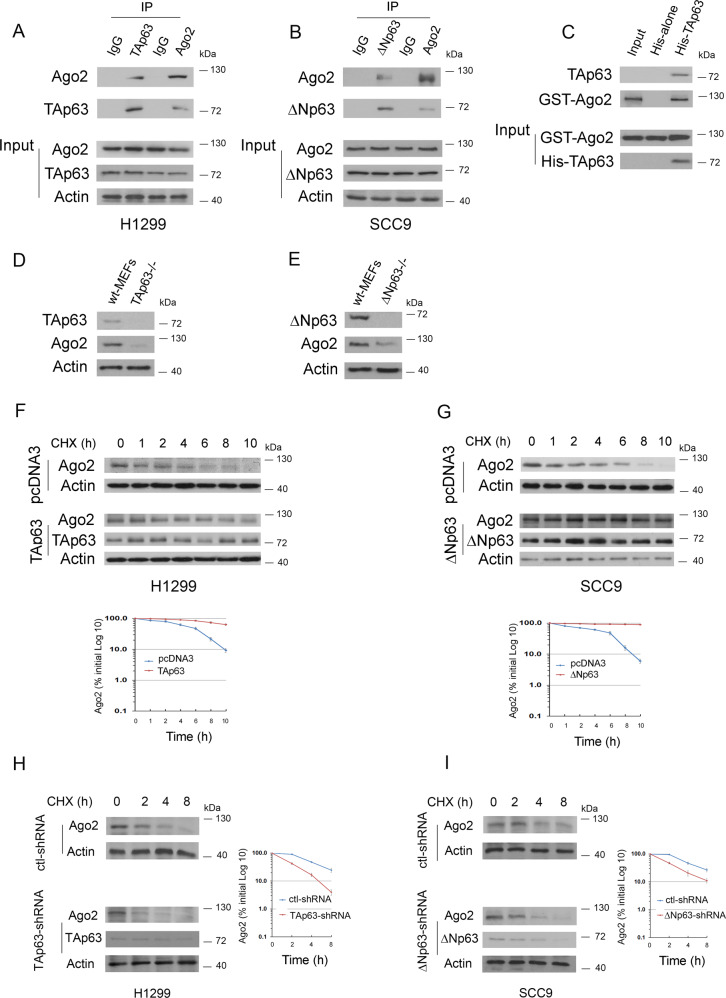
p63 interacts with and stabilizes Ago2. **A** Cell lysates were prepared from H1299 cells, immunoprecipitated with the indicated antibodies, and analyzed by Western blotting with antibodies to detect Ago2 or TAp63. Direct Western blots for Ago2, TAp63, and Actin are shown in the lower panels. **B** Similar to (**A**), except that SCC9 cells and ΔNp63 antibody were used. **C** In vitro interactions of TAp63 and GST-Ago2 were evaluated in Ni2^+^ pull-down assays. GST-Ago2 or His-TAp63 fusion proteins were purified from *E. coli*. The ability of GST-Ago2 to bind to His-TAp63 was analyzed by immunoblotting with an antibody against GST (GST-Ago2). **D** Cell extracts were prepared from TAp63^−/−^ and TAp63^+/+^ MEFs. Western blot analysis of endogenous Ago2 and TAp63 proteins with the indicated antibodies. **E** Cell extracts were prepared from ΔNp63^−/−^ and ΔNp63^+/+^ MEFs. Western blot analysis of endogenous Ago2 and ΔNp63 proteins with the indicated antibodies. **F**, **G** H1299 cells transfected with TAp63 (**F**) or SCC9 cells transfected with ΔNp63 (**G**) were treated with cycloheximide (CHX) (20 μg/ml) as indicated. Endogenous Ago2 levels were determined by immunoblotting. An antibody against β-actin was used as a loading control. The expression levels of Ago2 in H1299 cells (**F**) and SCC9 cells (**G**) were determined by densitometry of the immunoblots. Error bars indicate the SEM (*n* = 3). **H** H1299 clones stably expressing control-shRNA or TAp63-shRNA were treated with cycloheximide (20 µg/ml). Cells were harvested at the indicated time points. Endogenous TAp63 levels were measured by immunoblotting with a TAp63 specific antibody. An antibody against β-actin was used as a loading control. **I** Similar to (**H**), except that SCC9 clones and ΔNp63 specific antibodies were used. The expression level of Ago2 in H1299 (**H**) or SCC9 clones (**I**) was determined by densitometry of the immunoblots in (**H**) and (**I**). Error bars indicate SEM (*n* = 3).

We next sought to determine whether p63 isoforms interact with Ago2 under physiological conditions in cells. As shown in Fig. 2A, TAp63 coimmunoprecipitated with Ago2 in H1299 cells. Similarly, ΔNp63 coimmunoprecipitated with Ago2 in SCC9 cells (Fig. 2B). We then carried out an in vitro Ni2^+^ pull-down assay. GST-Ago2 bound to His-TAp63 but not to His alone, implying that this interaction is indeed direct (Fig. 2C). Notably, the basal levels of endogenous Ago2 protein were greatly decreased in TAp63^−/−^ mouse embryonic fibroblasts (MEFs) compared to parental wild-type (wt) MEFs (Fig. 2D). Similar data were obtained from ΔNp63^−/−^ MEFs (Fig. 2E). We next wanted to determine whether p63 isoforms modulate the stability of the Ago2 protein. The half-life of endogenous Ago2 was approximately 4 h in H1299 cells transfected with an empty vector (Fig. 2F, upper image), whereas it was approximately 8 h in the presence of TAp63 (Fig. 2F, lower image). The half-life of endogenous Ago2 was approximately >10 h in SCC9 cells transfected with ΔNp63 (Fig. 2G, lower image), whereas it was approximately 6 h in SCC9 cells transfected with an empty vector (Fig. 2G, upper image). The half-life of endogenous Ago2 in the presence of the control shRNA was approximately 4 h in H1299 cells; however, this half-life decreased to approximately 2 h when TAp63 was depleted (Fig. 2H). Similarly, the half-life of Ago2 decreased to approximately 2 h in ΔNp63-depleted SCC9 cells (Fig. 2I). The knockdown efficiency of TAp63 (H1299) and ΔNp63 (SCC9) was confirmed (Supplementary Fig. 2A, B). Together, our findings demonstrated that p63 isoforms regulate the stability of the Ago2 protein in cells.

### The effects of Ago2 on p63-mediated cell growth and survival analysis of Ago2 and p63 expression in several human cancers

**Fig. 3:**
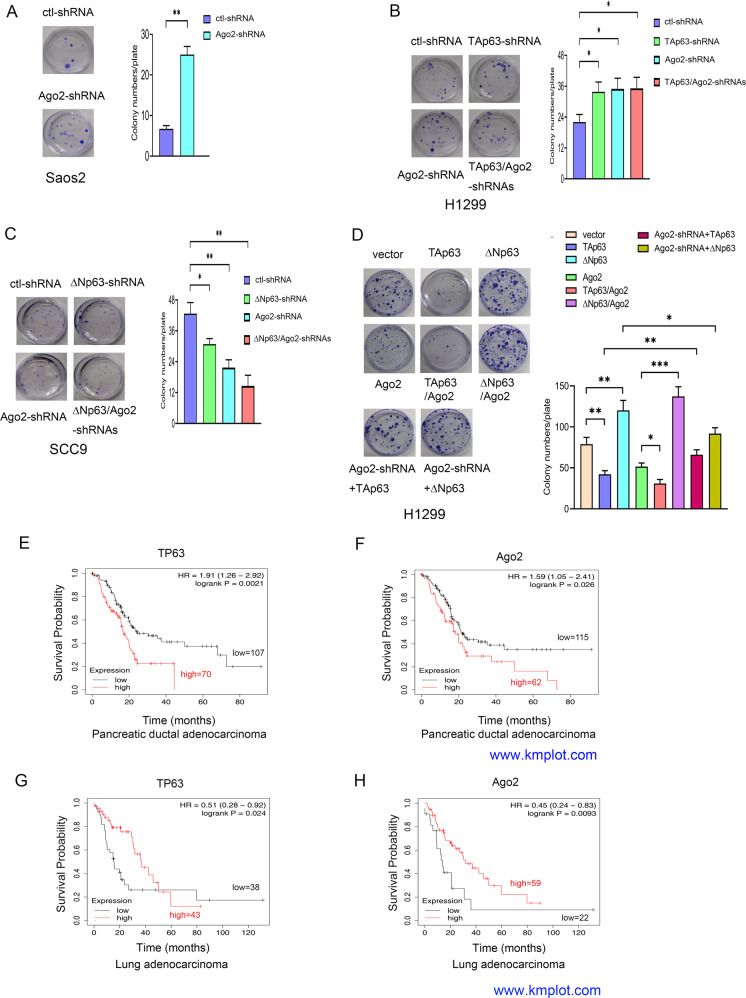
The effects of Ago2 on p63-mediated cell growth. The expression levels and prognosis of Ago2 and TP63 in human cancers. **A** The proliferative capability of Ago2- knockdown was tested through colony assays in Saos2 cells. Colony numbers were counted. **B**, **C** The proliferative capabilities of H1299 (**B**) and SCC9 cells (**C**) transfected with p63 shRNA or Ago2 shRNA alone or together were tested through colony assays as indicated. **D** The proliferative capability of H1299 cells transfected with TAp63, ΔNp63, or Ago2 alone or together with Ago2 was examined by colony assays. In addition, H1299 clones stably expressing Ago2-shRNA were transfected with plasmids expressing TAp63 or ΔNp63 as indicated. Colony numbers were countered and plotted. All experiments were performed in triplicate. *P < 0.05, **P < 0.01, ***P < 0.001. **E**, **F**. Kaplan–Meier survival curves revealed that patients with high levels of TP63 and Ago2 had a significantly lower overall survival in human pancreatic ductal adenocarcinoma (www.kmplot.com). **G**, **H** Kaplan–Meier survival curves generated from www.kmplot.com revealed that patients with high levels of TP63 and Ago2 had significantly better overall survival in human lung adenocarcinoma.

We further investigated the effects of Ago2 on cell growth when p63 isoforms were depleted or overexpressed. The proliferative capability of Saos2 cells was significantly increased after Ago2 depletion, suggesting that Ago2 suppresses cell growth as a tumor suppressor (Fig. 3A). H1299 cells markedly proliferated after depletion of TAp63 and/or Ago2 (Fig. 3B). In contrast, cell proliferation was inhibited by depletion of ΔNp63 and/or Ago2 in SCC9 cells (Fig. 3C). Cell proliferation was significantly inhibited when TAp63 and/or Ago2 were overexpressed, while the proliferative capability was significantly increased when ΔNp63 and/or Ago2 were overexpressed in H1299 cells (Fig. 3D). To determine whether TAp63 inhibits cell proliferation through the induction of Ago2, TAp63 or ΔNp63 expression constructs were transfected into Ago2-depleted H1299 cells. Ablation of Ago2 in H1299 cells impaired p63-dependent cell growth, implying that p63 inhibits or promotes cell proliferation partially through the induction of Ago2. Furthermore, p63 directly impacts Ago2 mRNA expression (Supplementary Fig. 2C). Next, we sought to determine whether there are differences in overall survival between TP63 and Ago2 expression in human cancers. Pancreatic ductal adenocarcinoma and colon cancer patients with high levels of TP63 and Ago2 had a significantly lower overall survival time (Fig. 3E, F, Supplementary Fig. 3A–D). However, lung adenocarcinoma patients with high expression of TP63 and Ago2 presented with better overall survival at early time points, but those with high levels of p63 had lower overall survival times at later time points, suggesting that overall survival might serve as a prognostic indicator of patients with these cancers (Fig. 3G, H).

### The regulatory effect of Ago2 on tumor angiogenesis is guided by p63 isoforms

**Fig. 4:**
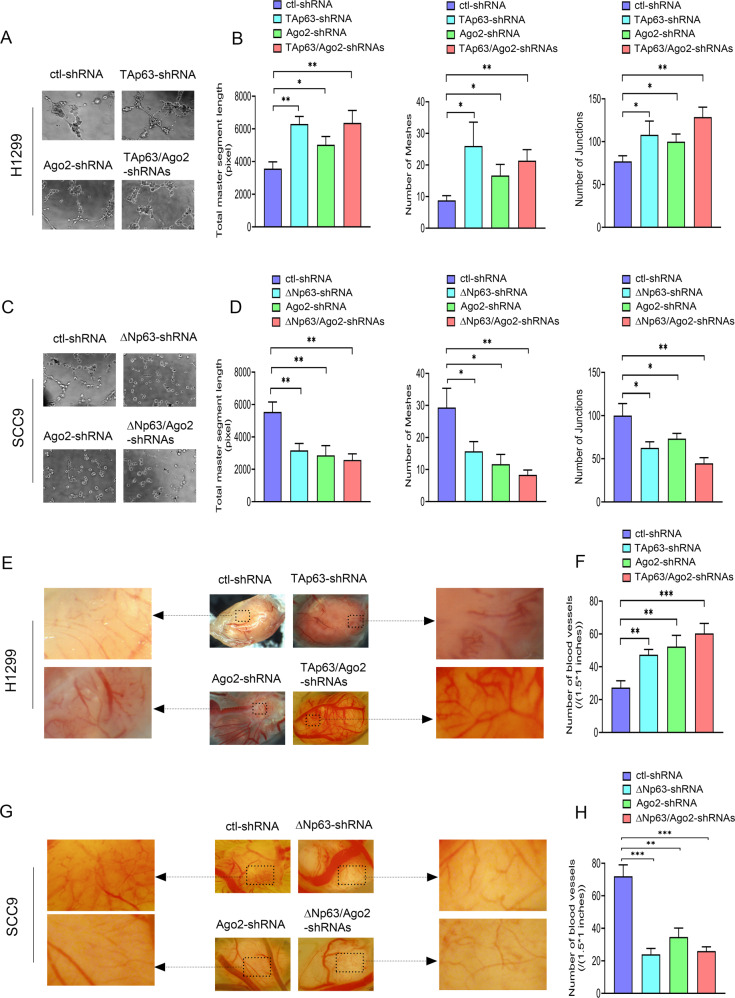
The regulatory effects of Ago2 on tumor angiogenesis are related to p63 isoforms in cells. **A**–**D** tube formation assay. **A**, **C** Representative images and (**B**, **D**) quantitative analysis of tube formation assay characterizing total master segment length, junction numbers, and mesh numbers in H1299 cells (**A**, **B**) or SCC9 cells (**C**, **D**) transfected with shRNAs for p63 or/and Ago2 as indicated. *n* = 3 per group. **E**–**H** CAM assays. **E**, **G** Representative images and (**F**, **H**) quantitative analysis of the CAM assay characterizing neovascularization in the chick CAM of H1299 clones (**E**, **F**) or SCC9 clones (**G**, **H**) expressing shRNAs for p63 or/and Ago2 as indicated. H1299 clones (5 × 10^6^ cells) or SCC9 clones (2 × 10^7^ cells) stably expressing shRNAs for p63 and/or Ago-2 were suspended in Matrigel and then inoculated onto the chick CAM. After 11 days, the primary tumors were imaged at 2X by a dissecting microscope. Images are representative of tumors from three separate experiments. Magnified images indicated by the arrow represent the typical vascular area (size: 1.5 × 1 inches in the original image (7.2 × 5.6 inches)) on tumors. Neovascularization was quantified by counting the number of capillaries of the typical vascular area (1.5 × 1 inches) on the tumor. All experiments were performed in triplicate. *p < 0.05; **p < 0.01; ***p < 0.001.

Angiogenesis is necessary for aggressive tumor growth and metastasis and constitutes an essential point in controlling cancer progression. When compared to the control cells, the ability of H1299 cells to induce HUVEC capillary tubule formation was significantly enhanced due to TAp63 and/or Ago2 knockdown (Fig. 4A, B); conversely, the ability of SCC9 cells to induce HUVEC capillary tubule formation was markedly attenuated due to ΔNp63 and/or Ago2 knockdown (Fig. 4C, D). Compared to the control cells, the ability of H1299 cells to induce HUVEC capillary tubule formation was significantly weakened due to TAp63 and/or Ago2 overexpression, while the capability of SCC9 cells to induce HUVEC capillary tubule formation was significantly promoted due to ΔNp63 and/or Ago2 overexpression (Supplementary Fig. 4A–D). Western blot visualized the expression of Ago2 and p63 proteins (Supplementary Fig. 4E, F).

We next tested whether Ago2, under the guidance of p63 isoforms, potentiates H1299 or SCC9 tumor progression in vivo. We used the standard chicken egg chorioallantoic membrane (CAM) assay for these studies [46, 47]. Compared to the control tumors, H1299 tumors with depletion of TAp63, Ago2, or both exhibited a significant increase in vascular density and vascular disruption, while SCC9 tumors with depletion of ΔNp63, Ago2, or both showed a substantial attenuation in vascular density and vascular disruption (Fig. 4E–H). Moreover, H1299 tumors that developed in the presence of Ago2 overexpression alone or with TAp63 exhibited much less neovascularization than the control H1299 tumors, while H1299 tumors that developed in the presence of Ago2 and ΔNp63 manifested higher vascular density and larger tumor size (Supplementary Fig. 4G). Strikingly, metastatic H1299 tumor nodules were found on the CAM in the presence of Ago2 and ΔNp63 (Supplementary Fig. 4G). Thus, ΔNp63 may induce a highly malignant transformation of Ago2 function and contribute to tumor dissemination. Ago2 and p63 protein levels were detected (Supplementary Fig. 4H). Taken together, our data indicated that different p63 isoforms could strongly guide Ago2 function in vitro and in vivo.

### miR-144 acts as a key regulator of the Itch-p63-Ago2 signaling pathways

**Fig. 5:**
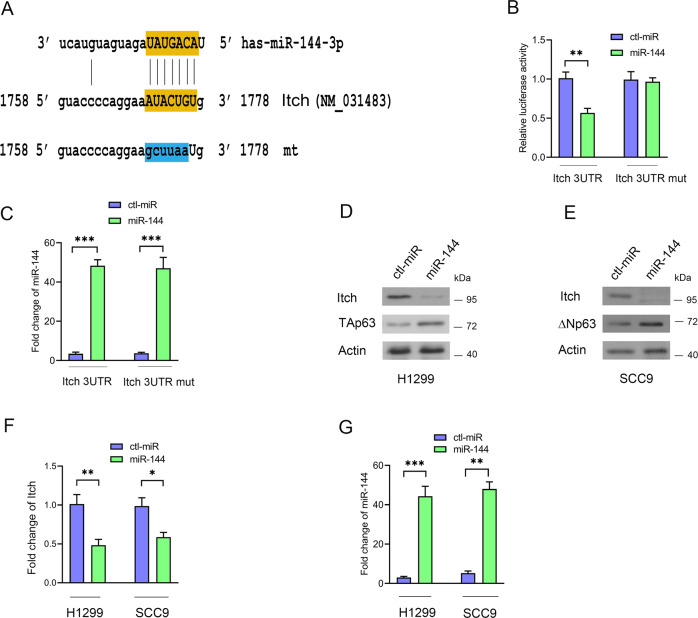
Itch is directly targeted by miR-144. **A** Predicted binding sites of miR-144 in the 3ʹUTR of Itch. The miR-144-3p seed region is shown. The mutated sequence (mt) was changed to lower-case letters. **B** H1299 cells were transfected with the indicated expression constructs. Luciferase activities were measured. **C** The levels of miR-144 expression were measured by qRT–PCR. **D**, **E** miR-144 negatively regulates Itch protein levels. Plasmids expressing control-miRNA or miR-144 were transfected into H1299 (**D**) or SCC9 (**E**) cells as shown. The levels of endogenous p63 and Itch protein were detected by Western blotting. Actin was used as a loading control. **F** The levels of Itch mRNA were measured by qRT–PCR in H1299 and SCC9 cells. **G** The levels of miR-144 expression were detected by qRT–PCR in H1299 and SCC9 cells. All experiments were performed in triplicate. *P < 0.05, **P < 0.01, ***P < 0.001.

To investigate miRNAs that regulate p63 activity, we designed a series of screening experiments [44, 45]. We selected forty miRNAs that may modulate p63 activity in tumor progression (Supplementary Fig. 5A). TAp63 protein levels increased with overexpression of miR-107, miR-144, miR-381, miR-34a, and miR-451 in H1299 cells (Supplementary Fig. 5B, C). miR-144/451 expression increased ΔNp63 protein levels in SCC9 cells (Supplementary Fig. 5D). We generated HeLa Tet-on clones that stably expressed three selected miRNAs. miR-144/451 induced TAp63 protein expression in HeLa Tet-on cells when the cells were treated with Dox (Supplementary Fig. 5E). Moreover, miR-144/451 strongly increased the activity of p21-Luc (Supplementary Fig. 5F). Itch is an E3 ubiquitin ligase for p63 [56]. We hypothesized that the miR-144/451 cluster targets the 3ʹUTR of Itch. The level of Flag-Itch2 protein was significantly decreased when cells were transfected with plasmids expressing Flag-Itch ORF with 3ʹUTR and miR-144; conversely, the levels of Flag-Itch protein were not altered when cells expressed Flag-Itch ORF and miR-144 or Flag-Itch ORF with 3’URT and control miRNA (Supplementary Fig. 6A, B). miR-144 expression was detected by qRT–PCR (Supplementary Fig. 6C). We found one putative MRE (miRNA response element) of miR-144 in the 3ʹUTR of human Itch by computational analysis (Fig. 5A, miRWalk2.0). Itch 3ʹUTR containing the putative binding site (WT) or mutation sites (mt, by site-directed mutagenesis) was cloned into pMIR-REPORT (Ambion). miR-144 significantly repressed the luciferase activity of the WT construct but not the mutation reporter, indicating that miR-144 reduces Itch protein by binding directly to the 3ʹUTR of Itch (Fig. 5B). miR-144 expression was measured by qRT–PCR (Fig. 5C). miR-144 overexpression resulted in an increase in TAp63 levels, which was accompanied by a decrease in endogenous Itch protein levels in H1299 cells (Fig. 5D). Similar results were obtained in SCC9 cells (Fig. 5E). We detected a decrease in the mRNA level of Itch in cells treated with miR-144 (Fig. 5F). The expression level of miR-144 was measured by qRT–PCR (Fig. 5G). Thus, Itch is a direct target of miR-144.

### The miR-144/451 cluster positively regulates p63 protein levels

**Fig. 6:**
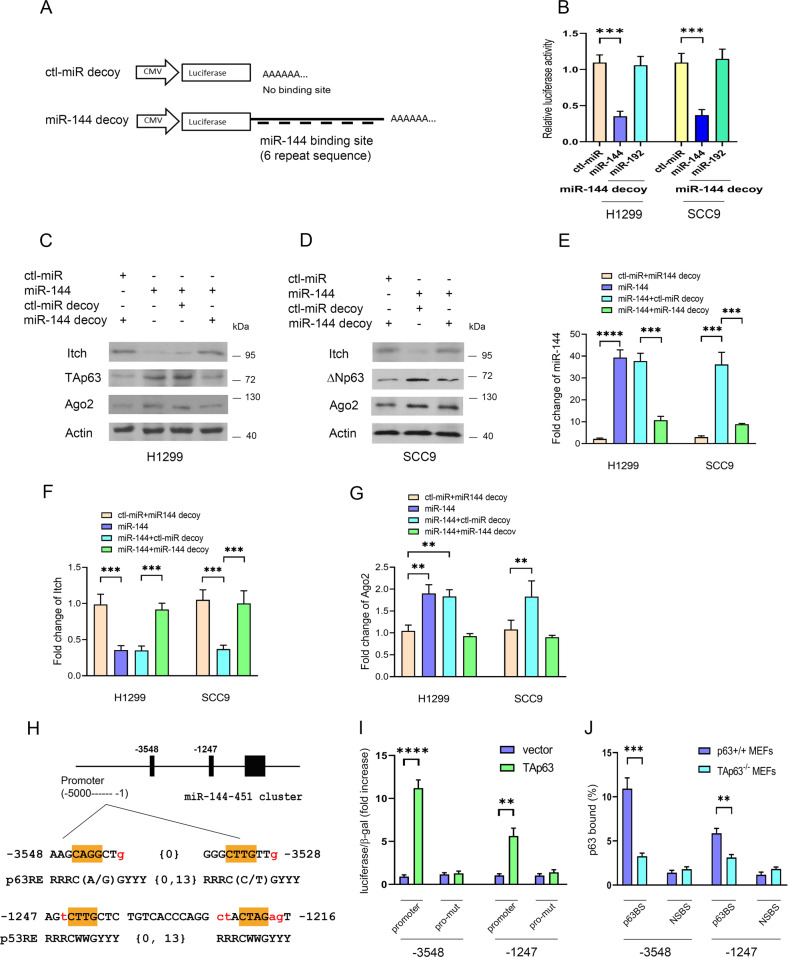
miR-144 regulates p63. **A** A miR-144 decoy was generated in which six tandem miR-144 sequences complementary to miR-144 were linked to a luciferase reporter gene. **B** Plasmids expressing control-miRNA, miR-144, miR-192, or in combination with miR-144 decoy were transfected into H1299 and SCC9 cells. Luciferase activities were measured. **C** Plasmids expressing control-miRNA, miR-144, control-miRNA decoy, and miR-144 decoy were transfected into H1299 cells. The levels of endogenous Itch, TAp63, and Ago2 proteins were detected by Western blotting as indicated. **D** Similar to (**C**), except that SCC9 cells were used. **E** The expression level of miR-144 was detected by the TaqMan^®^ method. **F** The level of Itch mRNA was detected by qRT–PCR. **G** The level of Ago2 mRNA was detected by qRT–PCR. **H** The two potential p63 binding sites in the promoter of the miR-144/451 cluster were compared to the consensus p63/p53 binding sites as indicated. **I** The relative luciferase activity in the presence of TAp63 or an empty vector is presented. The reporter containing wt or mutant sequences was examined in H1299 cells. **J** TAp63 ChIP assays, followed by qRT–PCR for the miR-144/451 cluster promoter using TAp63^+/+^ MEFs and TAp63^−/−^ MEFs, were performed. p63 binding was measured by qRT–PCR. TAp63BS: TAp63-binding site. NSBS: nonspecific binding site on the promoter of the miR-144/451 cluster. All experiments were performed in triplicate. **p < 0.01; ***p < 0.001; ****p < 0.0001.

To determine the functional role of endogenous miR-144, a miR-144 decoy was generated (Fig. 6A) [44, 45]. Luciferase activity was specifically reduced when the miR-144 decoy was coexpressed with miR-144 but not with control miRNA or miR-192, indicating that the miR-144 decoy was highly specific for miR-144 and was capable of binding to miR-144 (Fig. 6B). Itch protein was significantly reduced when miR-144 was overexpressed and restored when the miR-144 decoy was coexpressed with miR-144 (Fig. 6C, D). It is worth noting that the decrease in Itch was accompanied by an increase in the levels of endogenous p63 and Ago2 proteins. We verified miR-144 expression by qRT−PCR (Fig. 6E). We detected a decrease in the mRNA level of Itch in cells treated with miR-144 (Fig. 6F). Consistently, the level of Ago2 mRNA was increased (Fig. 6G). Interestingly, we identified two putative p53REs in the promoter of the miR-144/451 cluster: (1) the more distal (5ʹ) site at –3548 to –3528, which is the p63RE that contains different base pairs at the 5th and 16th positions compared to the p53RE (5ʹ-RRRC(A/G)(A/T)GYYYRRRC(A/T)(C/T)GYYY-3ʹ, [57, 58]). (2) The more proximal (3ʹ) site is at –1247 to –1216, which is p53RE (Fig. 6H). Luciferase expression from the reporter plasmids containing p63RE or p53RE was stimulated 11.2-fold or 5.6-fold, respectively, by TAp63 (Fig. 6I). Mutation of the promoter abolished their luciferase activity, confirming the specificity of p63 binding. We also investigated the binding ability of TAp63 to the miR-144/451 promoter by ChIP, and analyzed it by qRT–PCR (Fig. 6J). Whether ΔNp63 activates or represses the p21 promoter is still controversial [59, 60]. In this study, ΔNp63 failed to transactivate the promoter of the miR-144/451 cluster. miR-451 overexpression increased p63 protein levels (Supplementary Fig. 5B, 5E). However, we were unable to find an MRE for miR-451 in the 3ʹUTR of Itch. The miR-451 decoy was highly specific for miR-451 and was capable of binding to miR-451 (Supplementary Fig. 6D, 6E). When miR-451 was overexpressed in H1299 and SCC9 cells, the increase in p63 was accompanied by an increase in the level of endogenous Ago2 protein (Supplementary Fig. 6F, 6G). miR-451 expression was measured by qRT–PCR (Supplementary Fig. 6H). Taken together, our findings indicated that the miR-144/451 cluster regulates p63 protein levels.

### miR-144 induces apoptosis and suppresses cell invasion in a TAp63-dependent manner

**Fig. 7:**
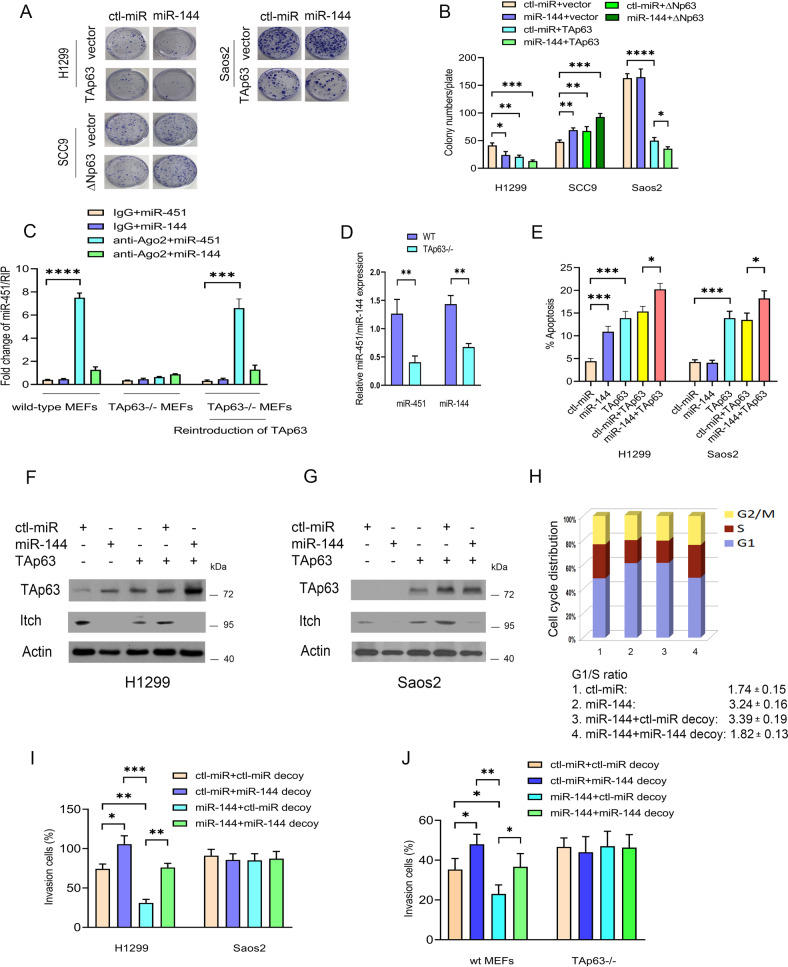
miR-144 acts as a key regulator of the Itch-p63-Ago2 pathway. **A** H1299, SCC9, and Saos2 cells were transfected with the indicated miRNA expression plasmids. A colony formation assay was performed. *n* = 3. **B** The numbers of colonies were counted and plotted in H1299, SCC9, and Saos2 cells, as indicated. **C** Ago2 RNA immunoprecipitation (RIP) was performed, followed by qRT–PCR. A RIP assay with an Ago2 antibody was used to assess the binding ability between miR-144/451 and Ago2 mRNA in wt MEFs, TAp63^−/−^ MEFs, or TAp63^−/−^ MEFs transfected with a TAp63 expression construct. Error bars represent the mean ± SD. All experiments were performed in triplicate. **D** The levels of miR-451 and miR-144 expression were detected by qRT–PCR. **E** H1299 and Saos2 cells were transfected with the indicated expression plasmids. The effect of miR-144 on TAp63-dependent apoptosis was determined by annexin V staining and flow cytometry. Error bars indicate the SEM. (*n* = 3). **F** Western blots were used to visualize the protein expression of TAp63 and Itch in H1299 cells. Actin was used as a loading control. **G** Similar to (**F**), except that Saos2 cells were used. **H** The effect of miR-144 depletion using miR-144 decoy on cell cycle arrest. H1299 cells were transfected with control-miRNA or miR-144, in combination with control miRNA decoy, or with miR-144 as indicated. Cell cycle analysis was determined by PI staining and flow cytometry. The results represent the average of triplicate experiments. The G1/S ratio is provided. *n* = 3. **I** H1299 and Saos2 cells were transfected with the indicated miRNA expression constructs. Transwell invasion (Matrigel matrix-coated) assays were performed. The numbers of invading cells were quantified using ImageJ software. **J** Wild-type MEFs or TAp63^−/−^ MEFs were transfected with the indicated miRNA expression constructs and subjected to Transwell invasion assays. All experiments were performed in triplicate. **P < 0.01, ***P < 0.001, ****P < 0.0001.

We examined the effect of miR-144 expression on cell proliferation. Cell proliferation was inhibited by miR-144 overexpression and was further decreased by coexpression of miR-144 and TAp63 in H1299 cells but not in SCC9 and Saos2 cells, implying that miR-144 enhanced TAp63-dependent growth suppression (Fig. 7A, B). miR-144 expression was measured by qRT–PCR (Supplementary Fig. 7A). The association of Ago2 with let-7 family miRNAs is significantly increased in response to Dox in the presence of wt-p53 but decreased in mutant p53 [50]. The binding of miR-451 to Ago2 was increased considerably compared to the IgG control antibody in wt MEFs but not in TAp63^−/−^ MEFs, and reintroduction of TAp63 into TAp63^-/-^ MEFs restored the association between miR-451 and Ago2 (Fig. 7C). miR-451/144 expression was measured by qRT–PCR (Fig. 7D). We next investigated the effect of miR-144 on TAp63-dependent apoptosis [44, 49]. miR-144 overexpression alone led to apoptosis; however, coexpression of miR-144 and TAp63 significantly increased apoptosis (Fig. 7E). Consistently, miR-144 overexpression did not induce apoptosis in Saos2 cells. Western blots were used to visualize TAp63 and Itch protein expression (Fig. 7F, G). miR-144 expression and Itch mRNA expression were detected by qRT–PCR (Supplementary Fig. 7B, C). Furthermore, the increase in apoptosis could be largely prevented by coexpression of miR-144 and miR-144 decoy in H1299 cells (Supplementary Fig. 7D). miR-144 expression was measured by qRT−PCR (Supplementary Fig. 7E). Together, these data demonstrated that miR-144 is involved in TAp63-dependent apoptosis.

**Fig. 8:**
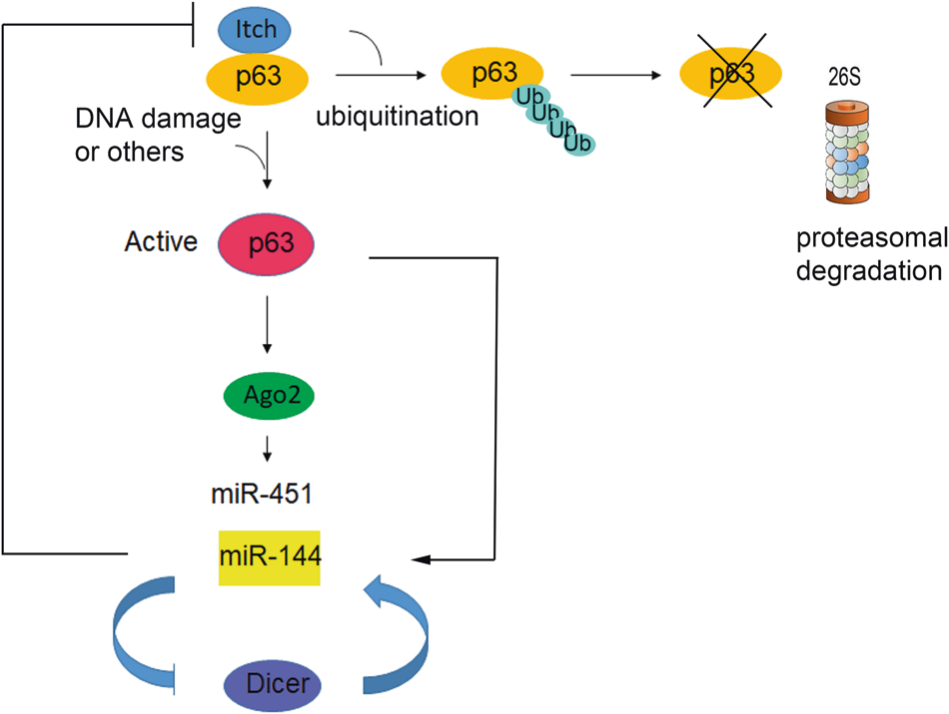
Model depicting miR-144 acts as a key regulator of the Itch-p63-Ago2/Dicer axis. p63 interacts with and activates Ago2. p63 strongly guides Ago2 dual functions. miR-144 activates TAp63 by targeting Itch, an E3 ligase of p63. TAp63 transactivates the promoter of the miR-144/451 cluster, forming a positive feedback loop. miR-144 targets Dicer by repressing global canonical miRNA processing and increases biogenesis in miR-451.

Cell cycle arrest mediated by TAp63 is vital for its tumor suppression function [17]. An increased change in the G1/S ratio is considered an indicator of G1 arrest. miR-144 overexpression increased the proportion of cells in G1 and decreased the percentage of cells in S phase, providing an increase in the G1/S ratio from 1.7 to 3.24; however, miR-144-induced G1 arrest was inhibited by coexpression of the miR-144 decoy (Fig. 7H). Moreover. miR-144 overexpression significantly inhibited the invasion of H1299 cells, while depletion of miR-144 produced the opposite effects (Fig. 7I). Critically, miR-144 overexpression did not affect the invasion of Saos2 cells, suggesting that the ability of miR-144 to suppress the invasion of cancer cells is TAp63-dependent (Supplementary Fig. 7F). The invasive capacity of wt MEFs was significantly increased when miR-144 was depleted by the miR-144 decoy, while miR-144 overexpression had no effect on the invasive ability of TAp63^-/-^ MEFs (Fig. 7J, Supplementary Fig. 7G). High expression of miR-144 and TP63 was found to be correlated with significantly better overall survival in breast cancer and bladder carcinoma, suggesting that miR-144 expression might have prognostic value for several human cancers (Supplementary Fig. 7H–K). We propose a model to elucidate the role of miR-144 in the Itch-p63-Ago2 pathways (Fig. 8).

## Discussion

TAp63 is a suppressor of tumorigenesis and metastasis. Delta Np63 (ΔNp63 or DNp63) lacks the N-terminal transactivation domain found in TAp63 and is considered an oncogene. Ago2 possesses endoribonuclease activity that is required for the canonical RNA interference mechanism [1–3]. Ago2 exhibits a dual function regulatory role in tumor progression. We observed that H1299 cells significantly proliferated after the knockdown of TAp63 and/or Ago2. In contrast, SCC9 cell proliferation was inhibited by knocking down ΔNp63 and/or Ago2. Depletion of Ago2 in H1299 cells impaired p63-mediated cell proliferation. This is the first report that p63 isoforms affect cell proliferation partially through the induction of Ago2 in H1299 cells.

As Ago2 is a common miRNA processing factor, increasing Ago2 levels may promote or repress tumor growth in patients with invasive carcinomas. Global increases in miRNA expression are seen in cases with high-risk multiple myeloma (MMs) with increased expression of Ago2 [9, 11]. In addition, Wu et al. observed that 25 miRNAs, including most let-7 family members and two miR-17-92 cluster members (miR-17a and miR-92-1), were upregulated, and seven miRNAs, including miR-145 and miR-361, were downregulated in Ago2-overexpressing MM cells [11]. In contrast, Zhang et al. observed that Ago2 overexpression inhibited tumor growth [13]. Here, we showed that p63 physically interacted with and activated Ago2. Ago2 collaborates with TAp63 or ΔNp63 to direct two different transcriptional programs that inhibit or promote cell proliferation and malignant transformation in HNSCC and NSCLC. Mechanistically, the seeming paradox regarding a tumor-suppressive versus oncogenic function of Ago2 and p63 isoforms can be reconciled in different tissue contexts. Previous data demonstrated a role for p63 isoforms in opposing or supporting oncogenic miR-155-mediated tumor growth and migration [61].

p63 isoforms bind and transactivate the Ago2 promoter through p53RE.ΔNp63 has a stronger binding capability to p53RE in the promoter of Ago2 than TAp63. Notably, ΔNp63 directly activates some target genes not induced by the TAp63 isoform [62]. Thus, p63 has the ability to regulate several genes with different effects and has opposite regulation depending on the isoform used. In addition, we observed a decrease in the expression level of the p63 isoforms after Ago2 was depleted. Depletion of Ago2 may enable some unknown miRNAs to target p63 isoforms or elevate E3 ligase(s) to reduce p63 isoforms. It is also possible that Ago2 is required for p63 stability. Ablation of Ago2 resulted in a decrease in p63 levels.

Kretov et al. showed that miR-144 targets Dicer in a negative feedback loop by repressing global canonical miRNA processing and activating Ago2-dependent miR-451 biogenesis [32]. Two questions have been raised: (1) Does miR-144 regulate Ago2 protein levels? (2) Do transcription factors regulate Ago2-mediated miR-451 biogenesis? miR-144/451 overexpression increased p63 protein levels. TAp63 transcriptionally activated the miR-144/451 cluster, forming a positive feedback loop. Furthermore, miR-144 activates TAp63 by directly targeting Itch, an E3 ligase of p63. We observed that the association between Ago2 and miR-451 is dependent on TAp63, suggesting that TAp63 plays a critical role in Ago2-mediated miR-451 biogenesis.

In conclusion, our study showed that p63 interacts with and activates Ago2. p63 plays a critical role in determining Ago2 dual functions. The miR-144/451 cluster increases p63 protein levels, and TAp63 transactivates the promoter of the miR-144/451 cluster. miR-144 enhances TAp63 tumor suppressor function and inhibits cell invasion. This is the first comprehensive report that miR-144 is a critical regulator of the Itch-p63-Ago2 pathway. This may lead to the development of novel therapies for the treatment of human tumors.

## Supplementary information

Supplementary Figures

Binder-original gels and blots for all figures

checklist- Reproducibility checklist

## Data Availability

The datasets used and analyzed during the current study are available from the corresponding author upon request. Original gels and blots for all figures are uploaded as ‘Supplemental Material’.
