# Supplementary material for: p63, a key regulator of Ago2, links to the microRNA-144 cluster
Source: Cell Death Dis. 2022 Apr 22;13(4):397. doi: 10.1038/s41419-022-04854-1 (PMC9033807; doi:10.1038/s41419-022-04854-1)
Supplement: Supplementary file 1 — Supplementary Figures [file 41419_2022_4854_MOESM1_ESM.pdf]

p63, a Key Regulator of Ago2, Links MicroRNA-144 Cluster

Benfan Wang[1*], H. Helena Wu[1*], Yasser Abuetabh[1], Sarah Leng[1], Sandra T. Davidge[2], Elsa R. Flores[3], David D. Eisenstat[4,5,6] and Roger Leng[1**]

[1]370 Heritage Medical Research Center, Department of Laboratory Medicine and Pathology, University of Alberta, Edmonton, Alberta T6G 2S2, Canada

[2]Department of Obstetrics & Gynecology & Physiology, 232 Heritage Medical Research Center, University of Alberta, Edmonton, Alberta T6G 2S2, Canada

[3]Department of Molecular Oncology, H. Lee Moffitt Cancer Center, 12902 Magnolia Drive, Tampa, FL 33612, USA

[4]Department of Oncology, Cross Cancer Institute, 11560 University Ave., University of Alberta, Edmonton, Alberta T6G 1Z2 Canada

[5]Department of Pediatrics, University of Alberta, 11405 - 87 Ave., Edmonton, Alberta T6G 1C9, Canada

[6]Murdoch Children's Research Institute, Department of Paediatrics, University of Melbourne, 50 Flemington Road, Parkville, Victoria 3052, Australia

Conflict of Interest

The authors declare no conflict of interest. *The first two authors contributed equally to this work.

**To whom correspondence should be addressed. rleng@ualberta.ca; Phone: (780) 492-4985.

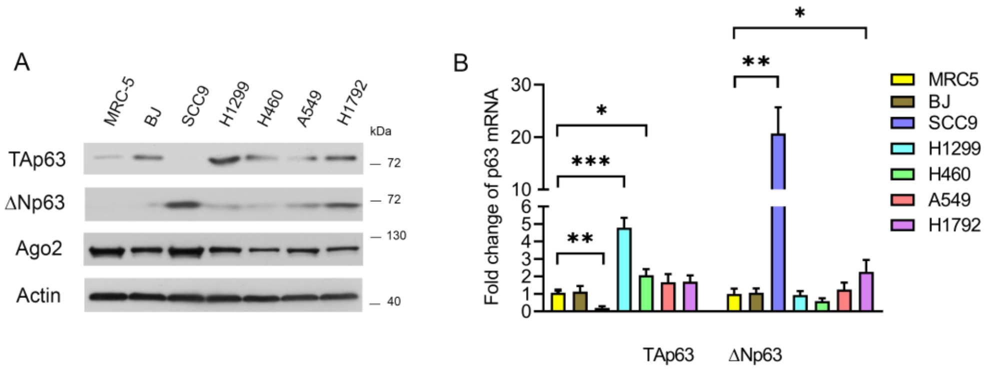

C

Probe ID	Links	p53 24h	p53R175H 24h	p73α 24h	p73β 24h	TAp63α 24h	ΔNp63α 24h	p53 6h	p53R175H 6h	p73α 6h	p73β 6h	TAp63α 6h	ΔNp63α 6h	REs
120032	RefSeq: NM_012154 HUGO: E1F2C2	–	–	–	–	– – –	–	–	↑ 1.72	–	–	↑ 1.49	–	HITS

– No statistically significant   – – – Negatively filtered   ↑ up regulated

Supplementary Fig. 1

Supplementary Fig. 1. Expression of p63 isoforms and Ago2 in cancer cell lines. (A) HNSCC cell lines and NSCLC cell lines were subjected to Western blotting to examine the expression of p63 isoforms and Ago2. An antibody against β-actin was used as a loading control. **(B)** mRNA levels of p63 isoforms in these cell lines were quantified by qRT-PCR as indicated. All experiments were performed in triplicate. **P* < 0.05, ***P* < 0.01, ****P* < 0.001. **(C)** Online Chip data showed that TAp63 upregulates the expression of Ago2 (about 1.5 fold).

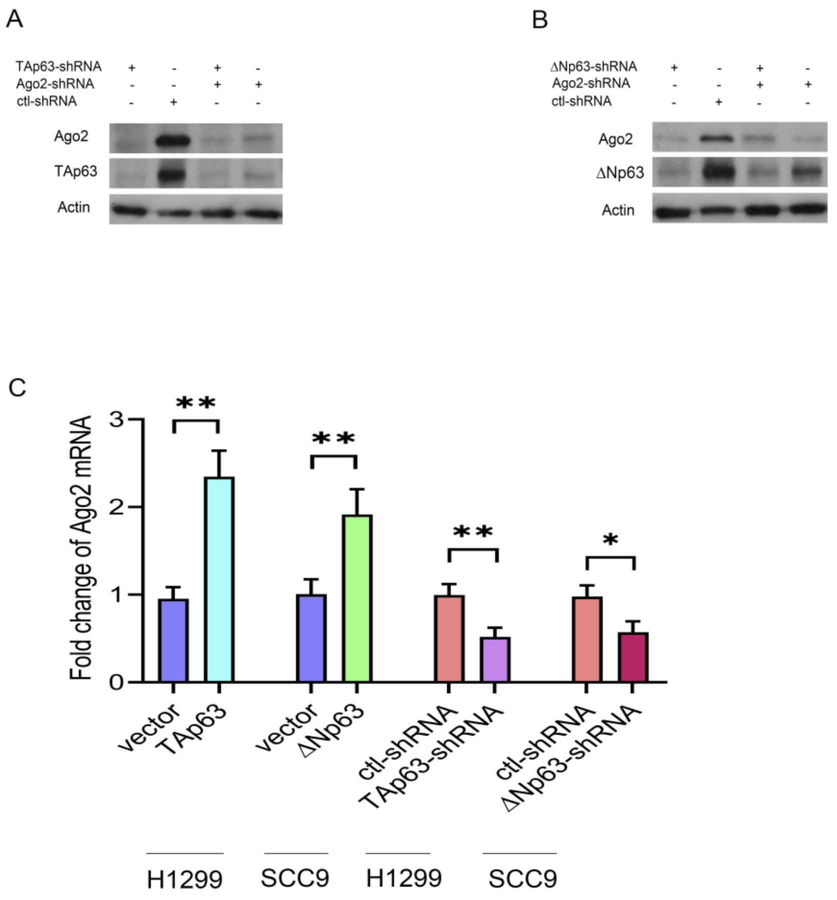

Supplementary Fig. 2

Supplementary Fig. 2. shRNA Ablation of p63 isoforms or Ago2. (**A, B**) Western blots visualize the efficiency of shRNAs for Ago2, TAp63, or ΔNp63 in H1299 cells (A) and SCC9 cells (B). **(C)** The expression level of Ago2 mRNA was detected by qRT-PCR when p63 isoforms were overexpression or knockdown as indicated. All experiments were performed in triplicate. $*P < 0.05$, $**P < 0.01$.

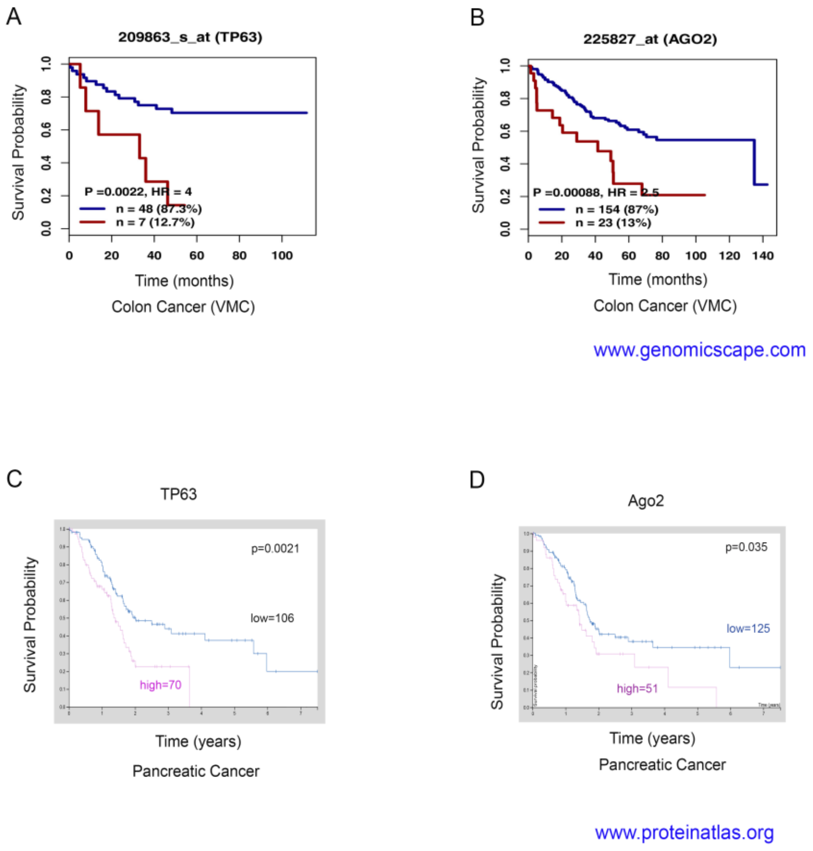

Supplementary Fig. 3

Supplementary Fig. 3. (A-B) Kaplan–Meier survival plotter analysis of TP63 (A) and Ago2 (B) in human colon cancer (www.genomicscape.com). **(C-D)** Kaplan–Meier survival plotter analysis of TP63 (A) and Ago2 (B) in human pancreatic cancer (www.proteinatlas.org).

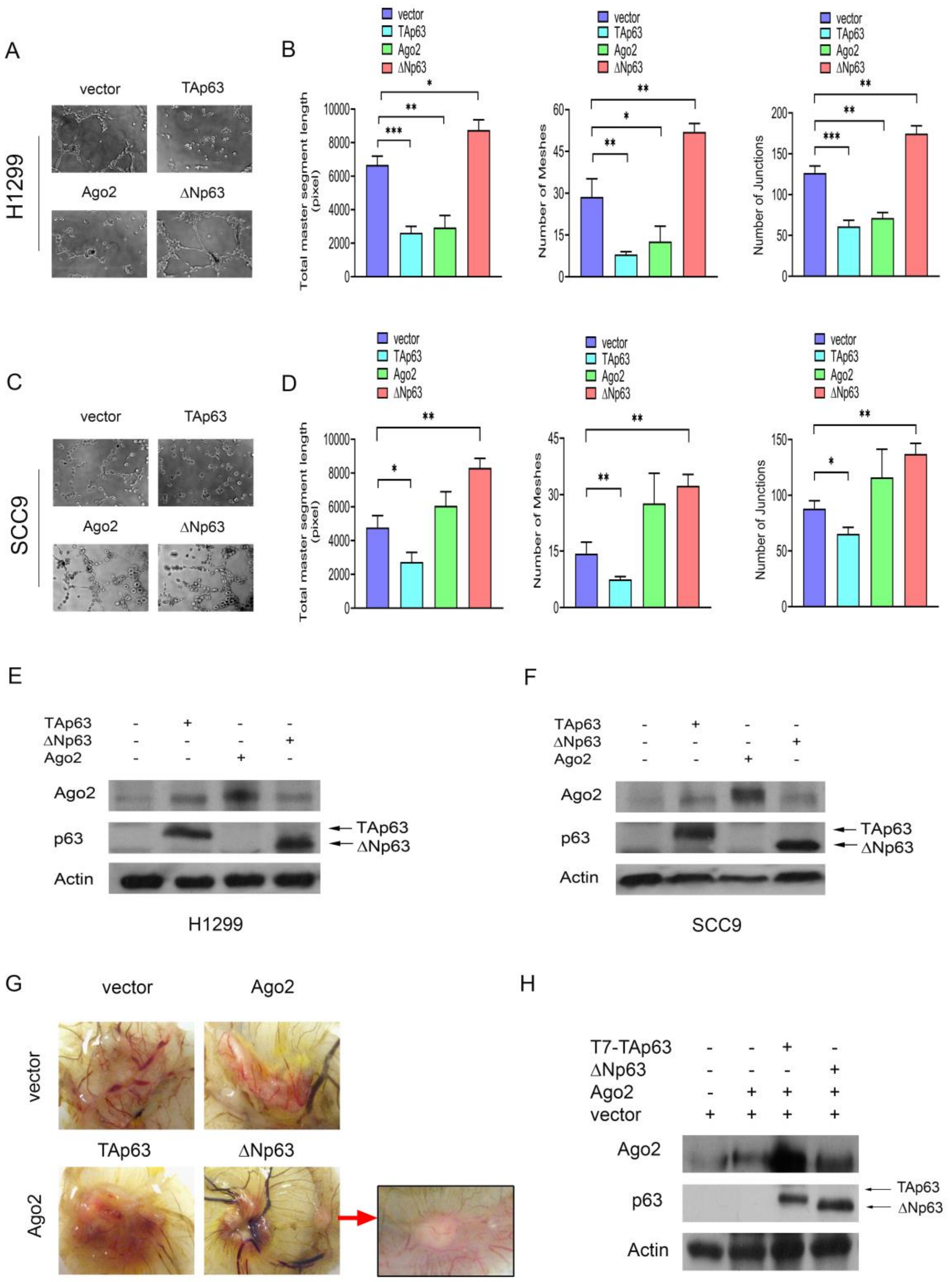

Supplementary Fig. 4

Supplementary Fig. 4. The regulatory effect of Ago2 on angiogenesis under the guidance of p63 isoforms. (A-D) Representative images **(A, C)** and quantitative analysis **(B, D)** of tube formation assay characterizing total master segment length, junction numbers, and mesh numbers in H1299 stable clones **(A, B)** or SCC9 stable clones **(C, D)** expressing TAp63, ΔNp63, and Ago2 as indicated. n = 3 per group. * *p* <0.05; ***p* <0.01; ****p* <0.001. **(E, F)** Western blots visualize the expression of Ago2, TAp63, and ΔNp63 in H1299 clones (E) or SCC9 clones (F). **(G)** Representative images of CAM assay characterizing neovascularization in the chick CAM of H1299 clones stably expressing Ago2 alone or with p63 isoforms as indicated. H1299 clones (5×10^6 cells) expressing Ago2 alone or p63 isoforms were suspended in matrigel then inoculated onto the chick CAM. After 11 days, the primary tumors were taken photos. Magnification images indicated by the arrow represent the typical migratory tumor nodules on the chick CAM. **(H)** Western blots visualize expressions of endogenous Ago2, ectopic TAp63, or ΔNp63 in H1299 stably expressing Ago2 alone or TAp63 or ΔNp63 as indicated.

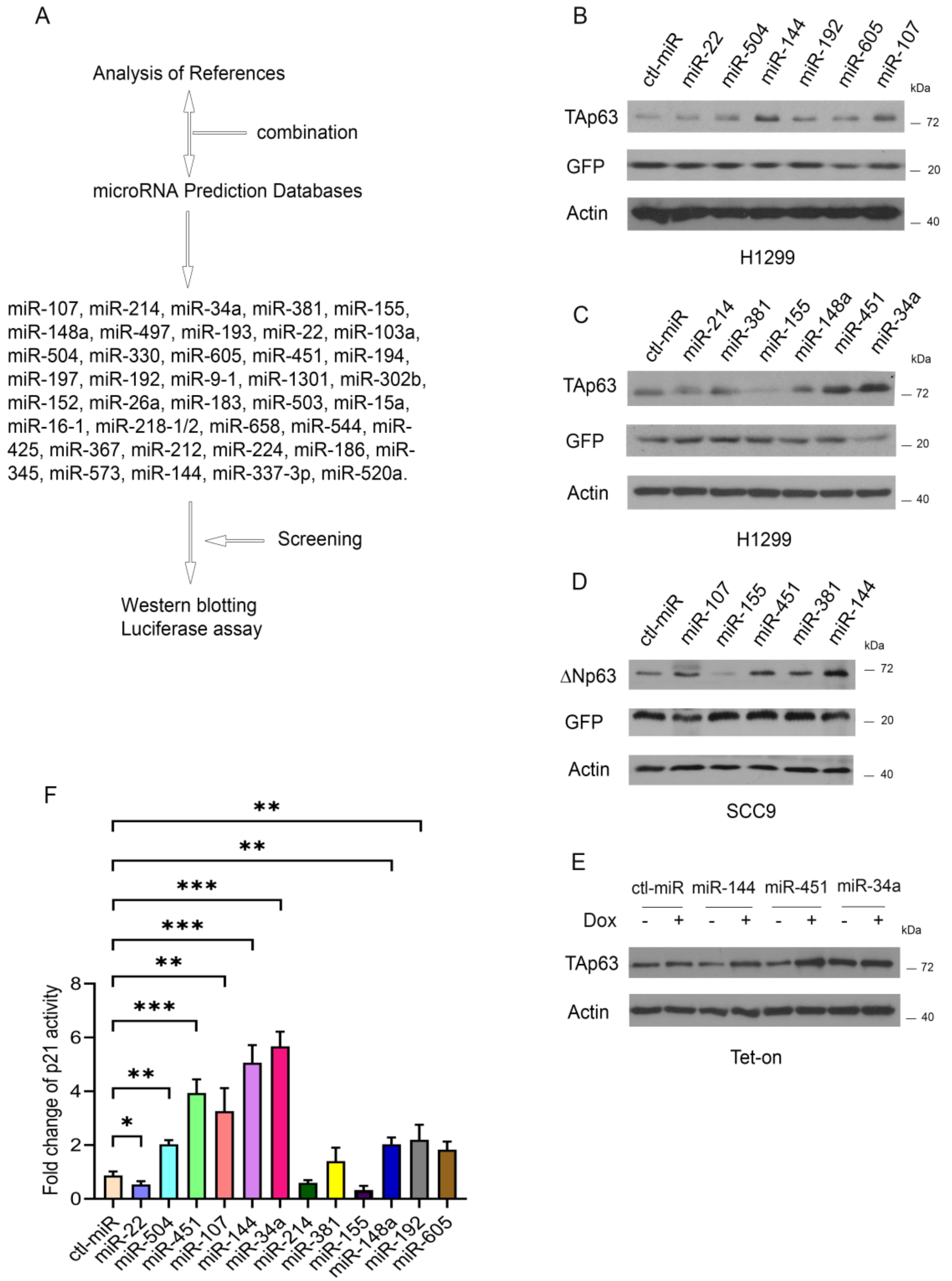

Supplementary Fig. 5

Supplementary Fig. 5. Screening for miRNAs that regulate TAp63 expression. (A) Summary of the screen for miRNAs that modulate TAp63 expression. **(B, C)** H1299 cells were transfected with the plasmids expressing various miRNAs as indicated. The TAp63 protein levels were detected by Western blotting. **(D)** Similar to (B, C), except that SCC9 cells and ΔNp63 specific antibodies were used as indicated. **(E)** The selected miRNA precursors were transfected into HeLa Tet-on inducible cell lines. The cells were treated with Dox (2 mg/ml). Western blots analyzed the levels of TAp63 protein. **(F)** The selected miRNAs and a p21-Luc reporter were co-transfected into H1299 cells. Luciferase activities were measured. The expression level of GFP was used to monitor transfection efficiency. Error bars indicate SEM (n =3). *p <0.05; **p <0.01; ***p <0.001.

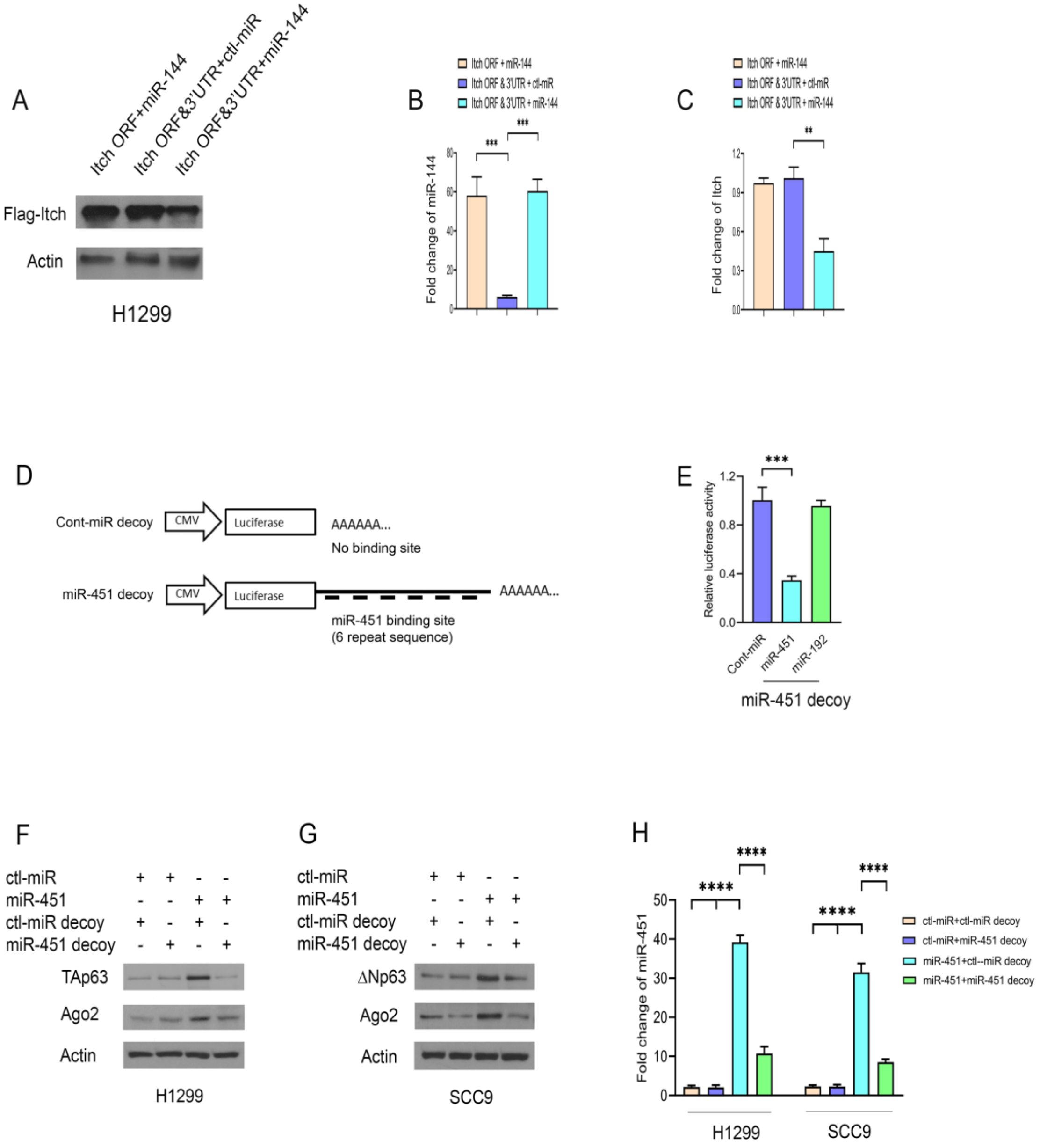

Supplementary Fig. 6

Supplementary Fig. 6. Itch is a direct target of miR-144. (A) Plasmids expressing Itch ORF, Itch ORF with 3'UTR, control-miRNA, miR-144 were cotransfected into H1299 cells as indicated. The levels of exogenous Itch (Flagged) were detected by Western blotting. **(B)** The levels of miR-144 expression were detected by qRT-PCR. **(C)** The levels of Itch mRNA expression were measured by qRT-PCR. **(D)** The structure of the miR-451 decoy. **(E)** miR-451 decoy specifically suppressed miR-451 luciferase activity, not with miR-192 (right). Plasmids expressing control-miRNA, miR-451, miR-192 were transfected into H1299 cells. Luciferase activity was measured. **(F)** Plasmids expressing miR-451 or miR-451 decoy were transfected into H1299 cells. The levels of endogenous TAp63, and Ago2 proteins were measured by Western blotting. (**G**) Similar to (F), except that SCC9 cells were used. **(H)** The levels of miR-451 were detected by qRT-PCR in H1299 and SCC9 cells, as indicated. **$p < 0.01$; ***$p < 0.001$; ****$p < 0.0001$. (n=3). All results are from three independent experiments.

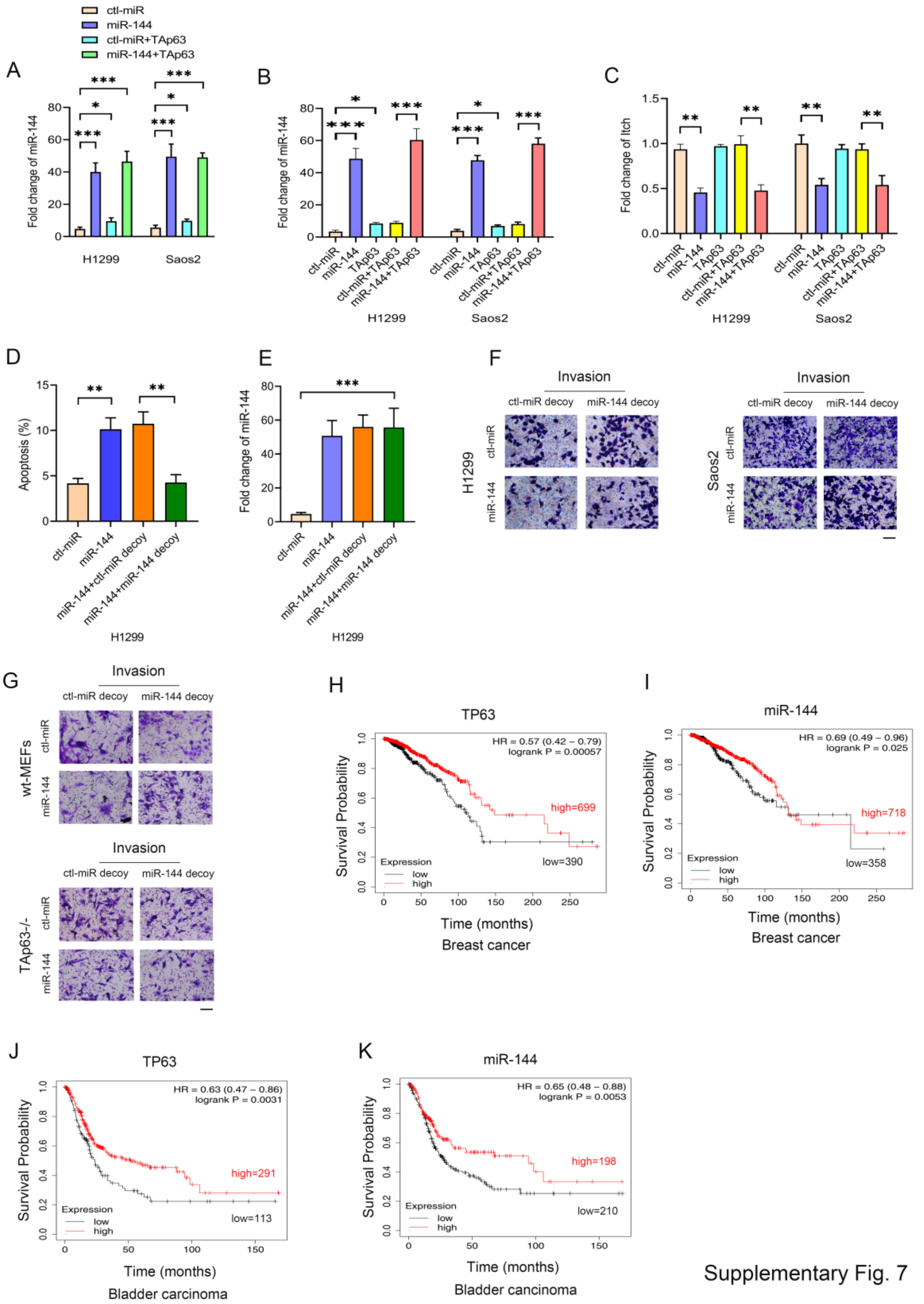

Supplementary Fig. 7

Supplementary Fig. 7. The role of miR-144 in tumor progression. **(A)** H1299 and Saos2 cells were transfected with the indicated expression plasmids. A colony assay was performed. The level of miR-144 expression was detected by qRT-PCR. **(B)** H1299 and Saos2 cells were transfected with the indicated expression plasmids. The effect of miR-144 on TAp63-dependent apoptosis was determined by annexin V staining and flow cytometry. The levels of miR-144 expression were detected by qRT-PCR. **(C)** The level of Itch mRNA expression was measured by qRT-PCR. **(D)** Similar to (B), H1299 cells were transfected with plasmids expressing control-miRNA, miR-144, or in combination with control miRNA decoy or miR-144 decoy as indicated. Apoptosis assay was performed. **(E)** The levels of miR-144 expression were measured by qRT-PCR. (**F**) Digital photomicrographs of invasion cells as indicated. All images are representative of three independent experiments at a magnification of 200x. All experiments were performed in triplicate. *$P < 0.05$, **$P < 0.01$, ***$P < 0.001$. **(G)** Representative images in MEFs cells are presented as indicated. Scale bar: 50 μM. **(H-K)** Kaplan-Meier curves for overall survival in several human cancer patients were determined using online databases as indicated (www.kmplot.com) in human breast cancer: (**H**) TP63, (**I**) hsa-miR-144, and in human bladder cancer (**J**) TP63, (**K**) hsa-miR-144.

Supplementary Table 1. Prediction of p63 binding sequence in Ago-2.

No.	Sequence	CHR_START (-1)	Mismatch Base	Name
>ENSG00000123908_1	gtggaagact cagtgca aaccttgttc tgaggaag gcacatgctc	141695323	2	intron-1
>ENSG00000123908_2	agtcttgctc tgttgcct aggctagagt gcag tggcacgatc	141697552	3	intron-2
>ENSG00000123908_3	agacttgctc cc gagcctgcgc cac agcccatccc	141698498	2	intron-3
>ENSG00000123908_4	catccagccc aaggag gggcaagtgt gggagac aggcaggccc	141700473	2	intron-4
>ENSG00000123908_5	agacatgtgc caccatgccc agc gaaggtgttt	141710179	3	intron-5
>ENSG00000123908_6	cagcaagggc tgg agacatgtca cagcaagcac	141717172	3 or 4	promoter-1
>ENSG00000123908_7	aaagctgctt t aatcatgtgc a gaacatgttt	141717562	2	promoter-2
