# Supplementary material for: p63, a key regulator of Ago2, links to the microRNA-144 cluster
Source: Cell Death Dis. 2022 Apr 22;13(4):397. doi: 10.1038/s41419-022-04854-1 (PMC9033807; doi:10.1038/s41419-022-04854-1)

Figure 1A

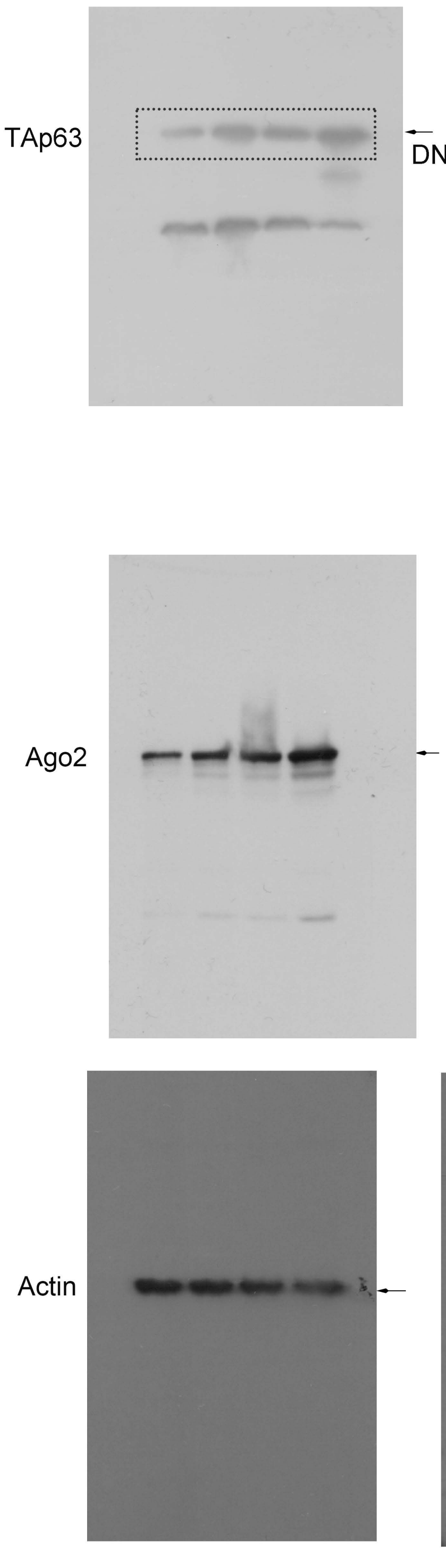

Figure 1B

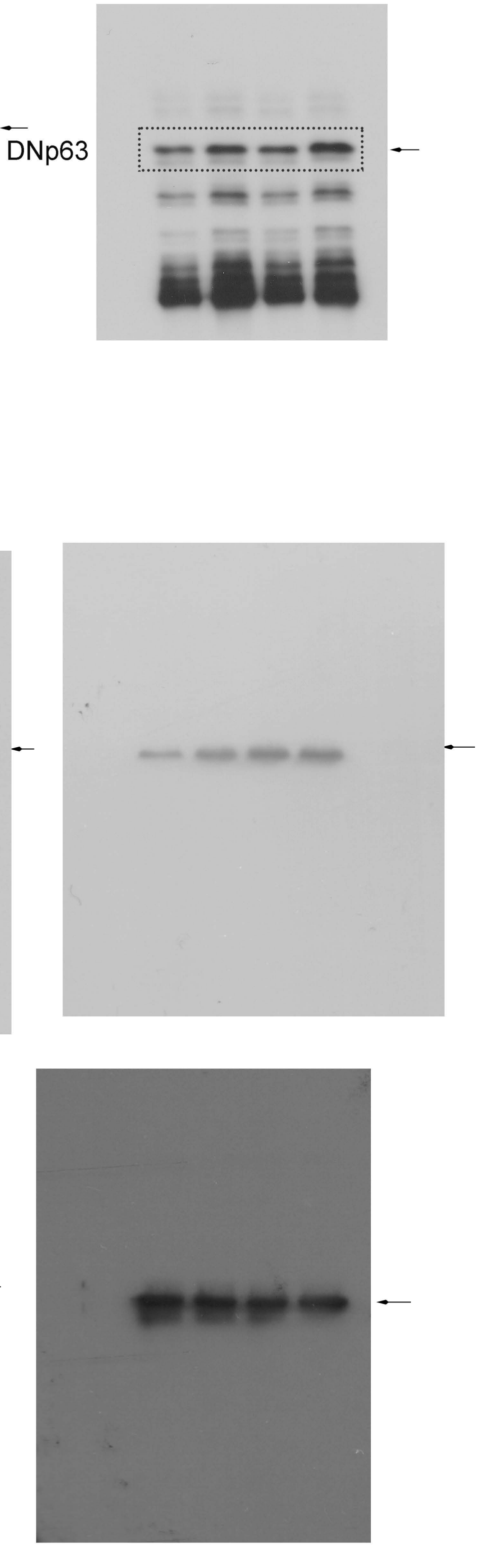

Figure 1F-G

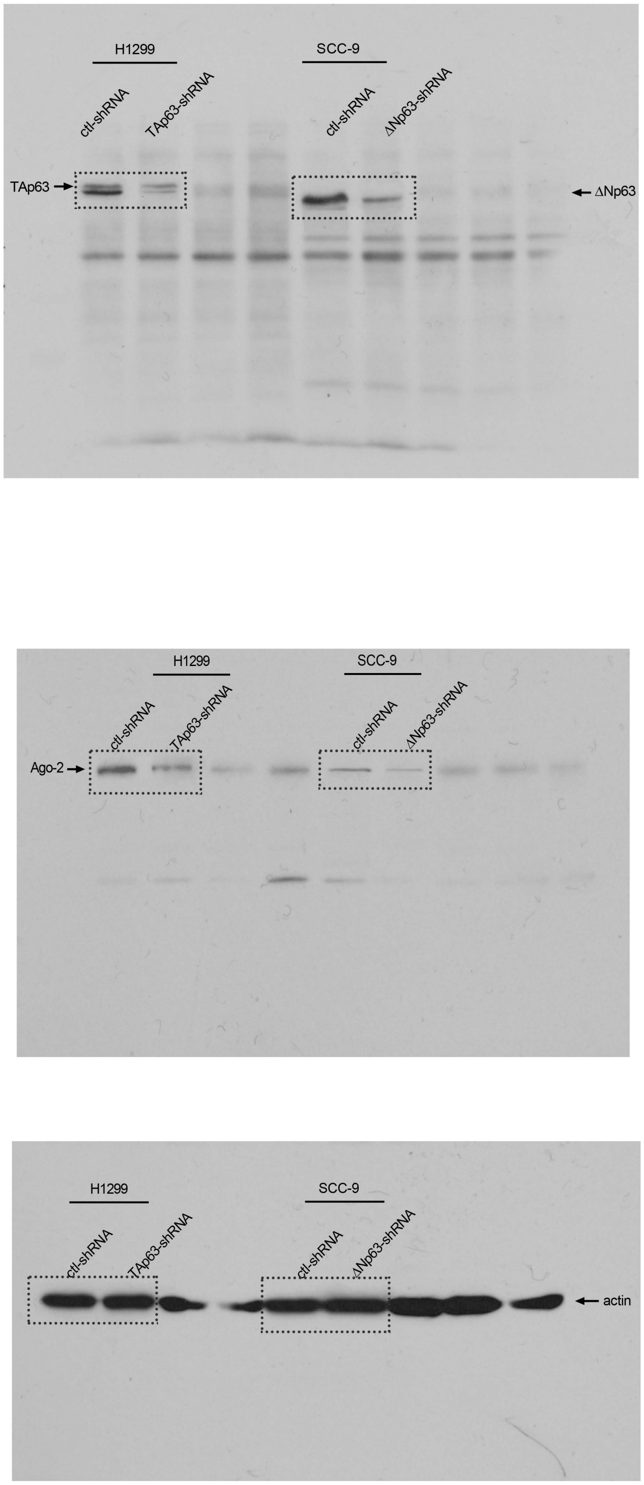

Fig-2A

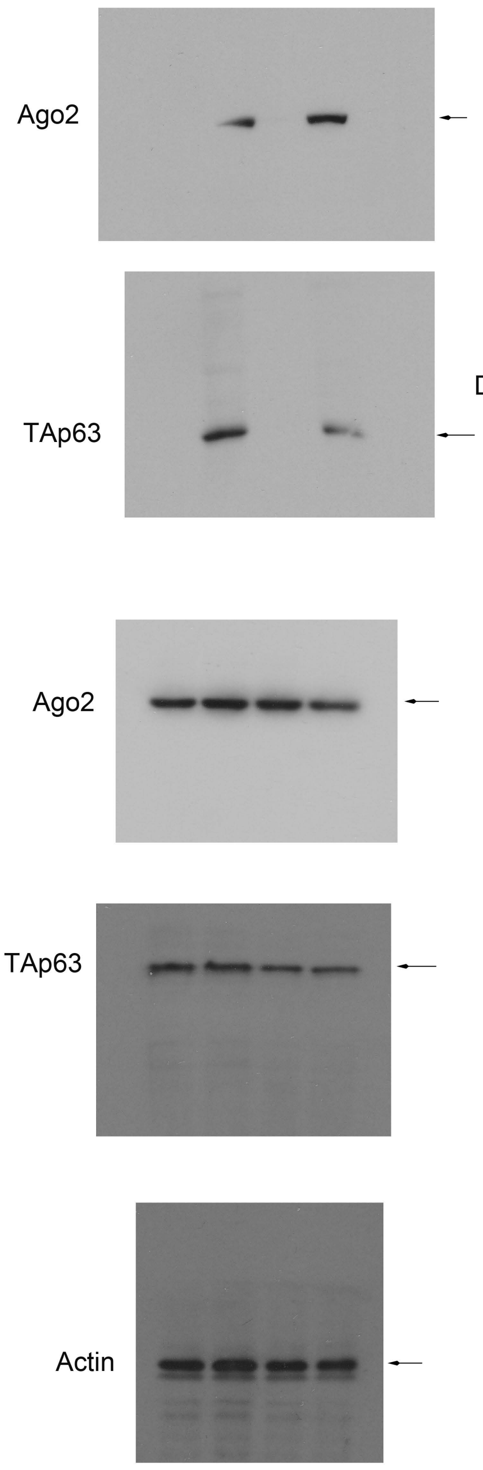

Fig-2B

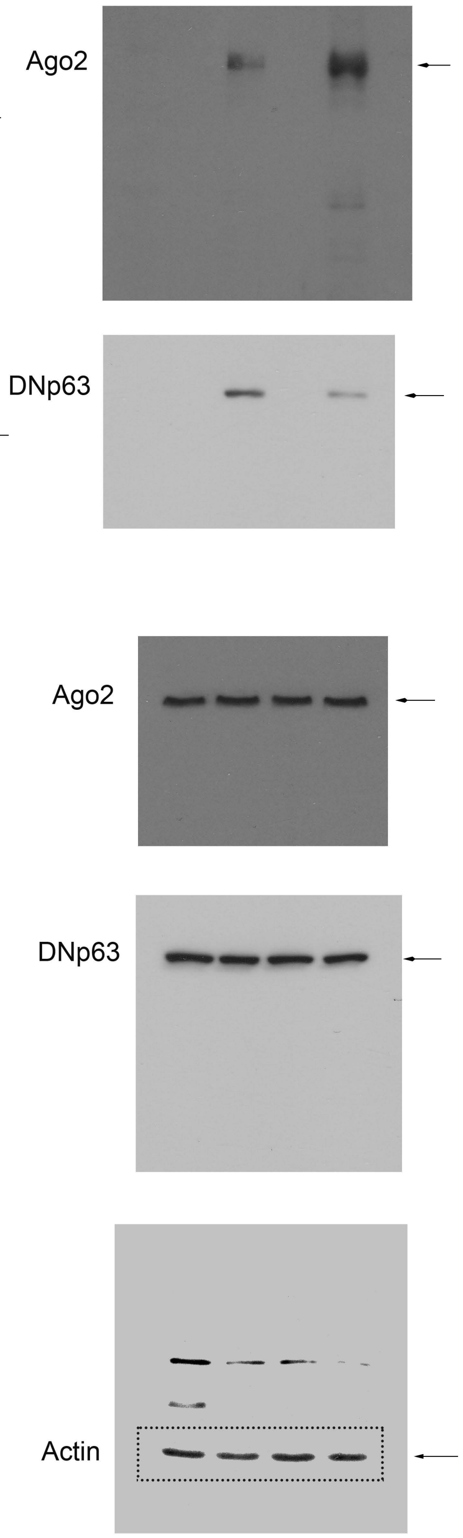

Fig-2C

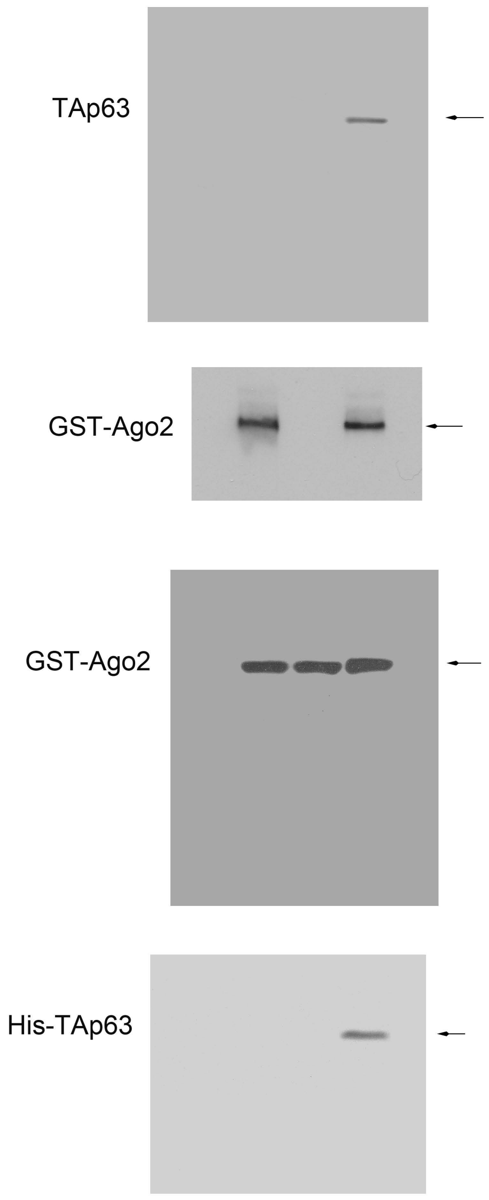

Figure 2D

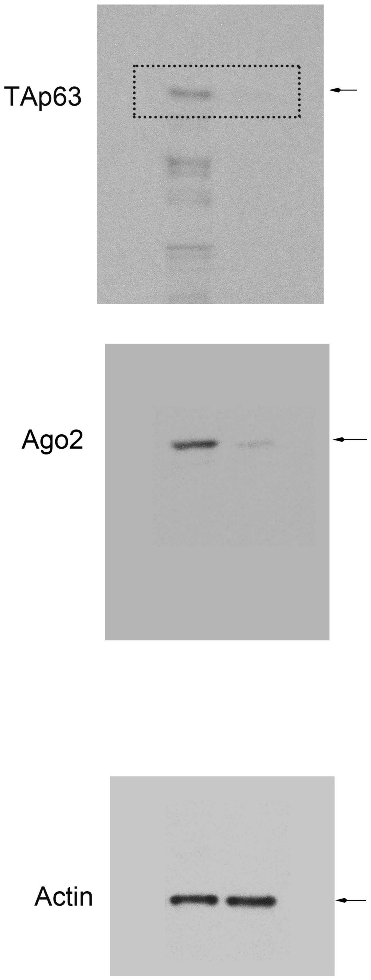

Figure 2E

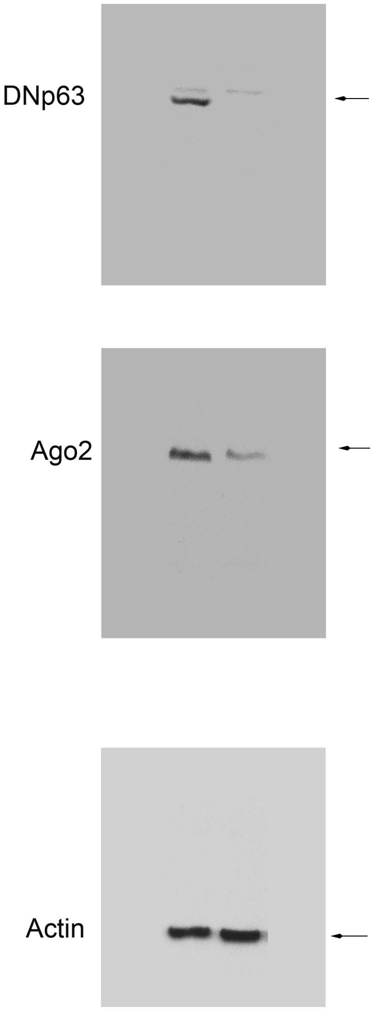

Figure 2 F

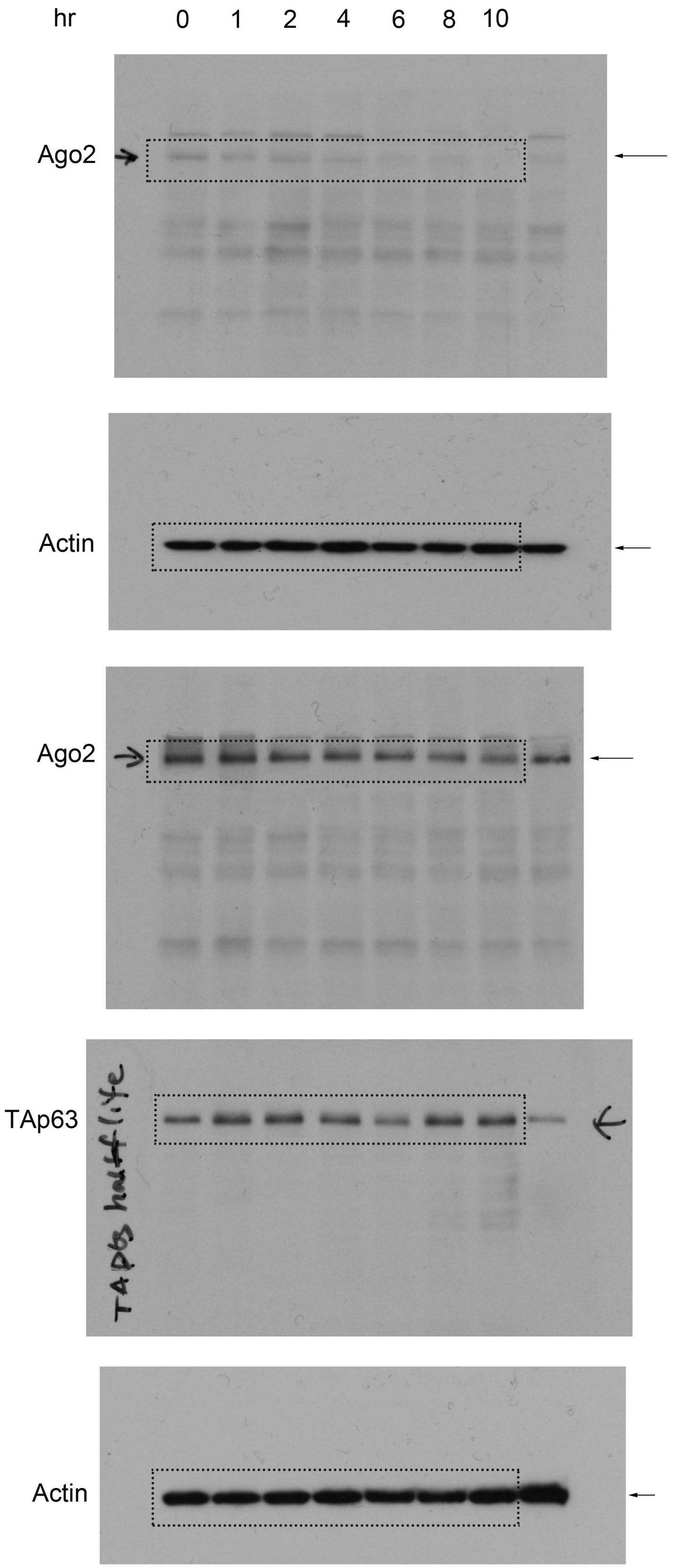

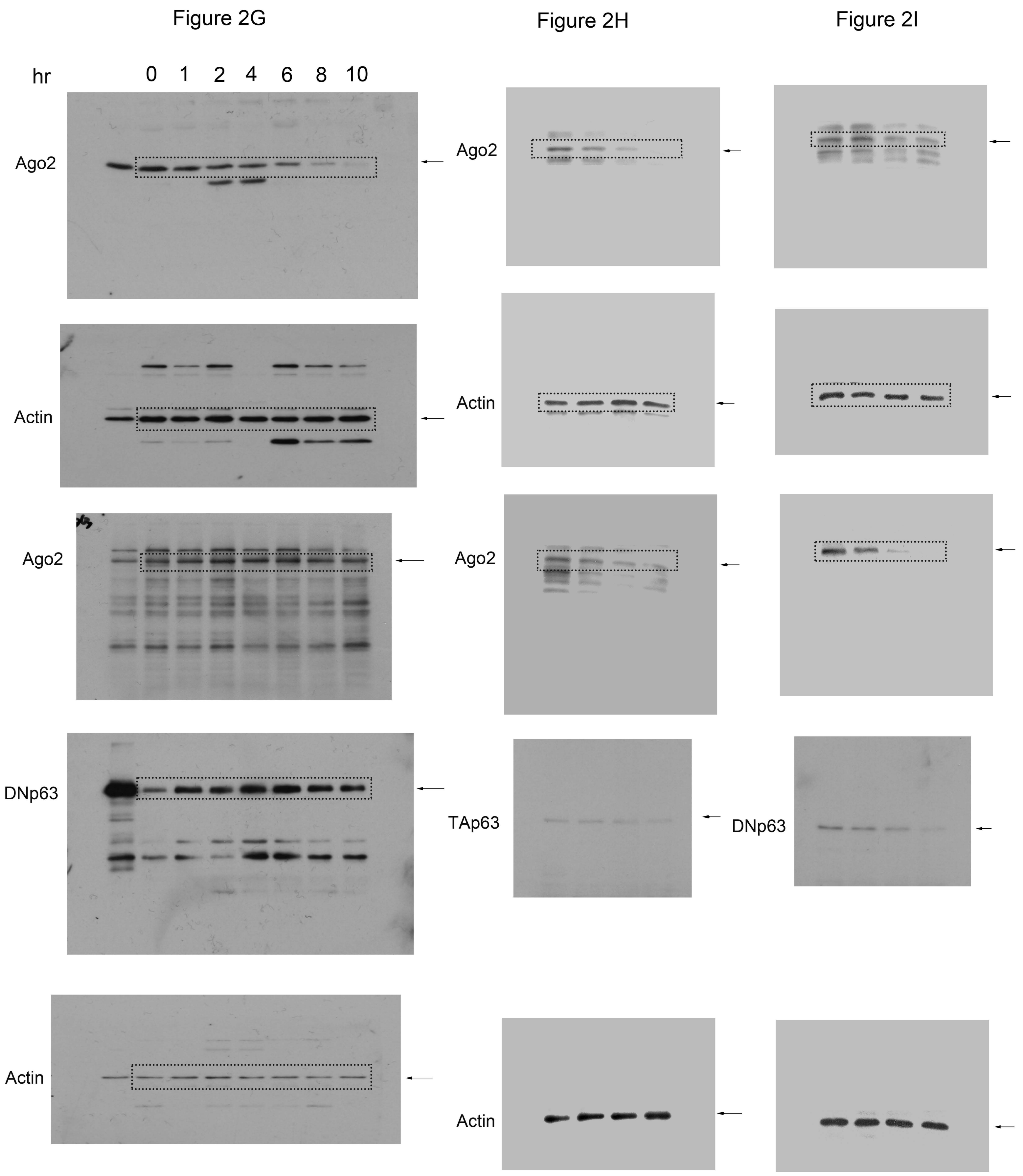
Figure 2G
Figure 2H
Figure 2I
hr
0 1 2 4 6 8 10
Ago2
Ago2
Ago2
Actin
Actin
Actin
Ago2
Ago2
Ago2
DNp63
TAp63
DNp63
Actin
Actin
Actin

Figure 5D

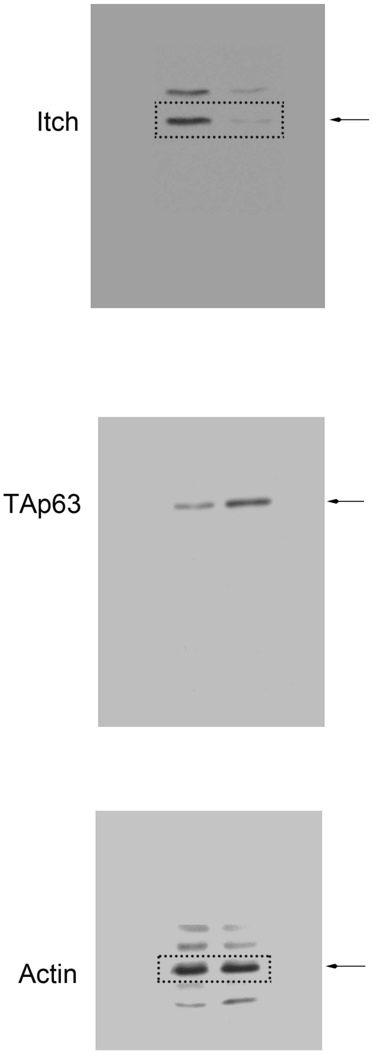

Figure 5E

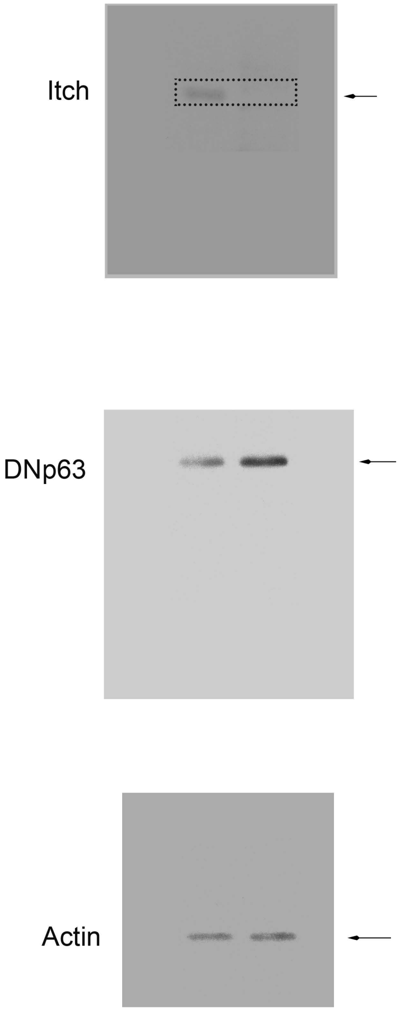

Figure 6C

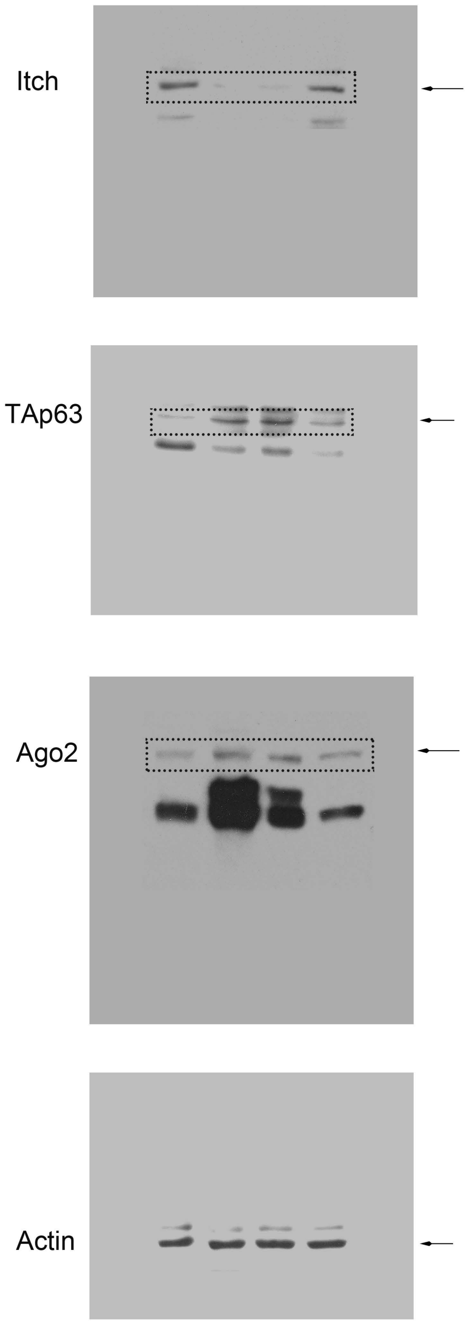

Figure 6D

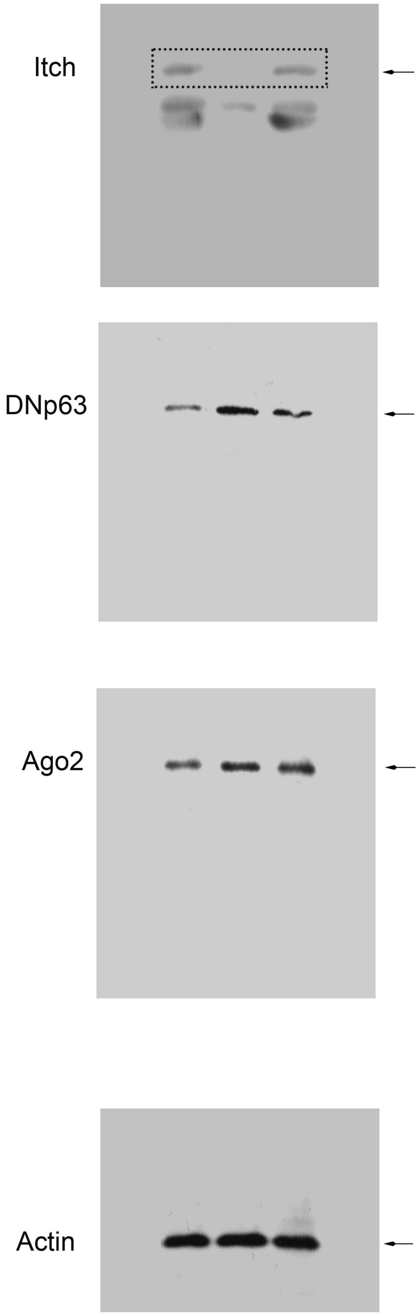

Figure 7F

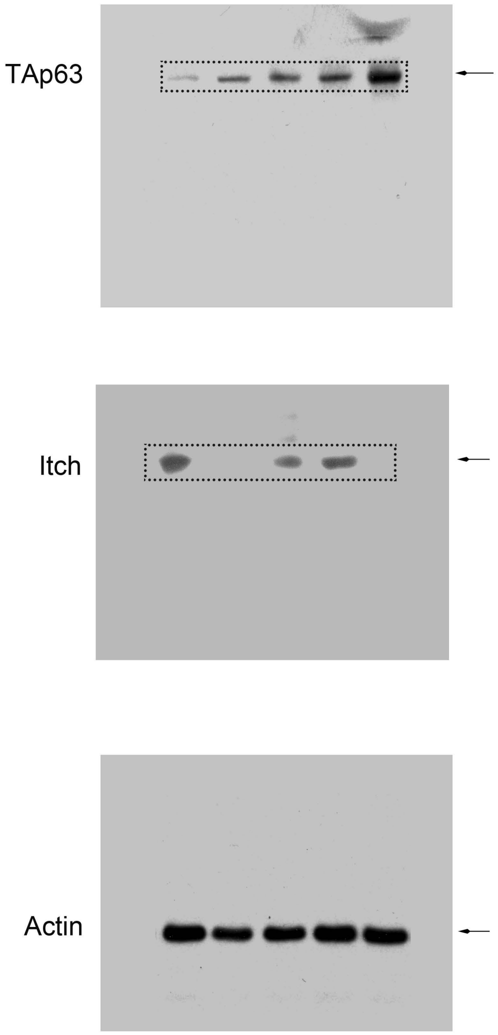

Figure 7G

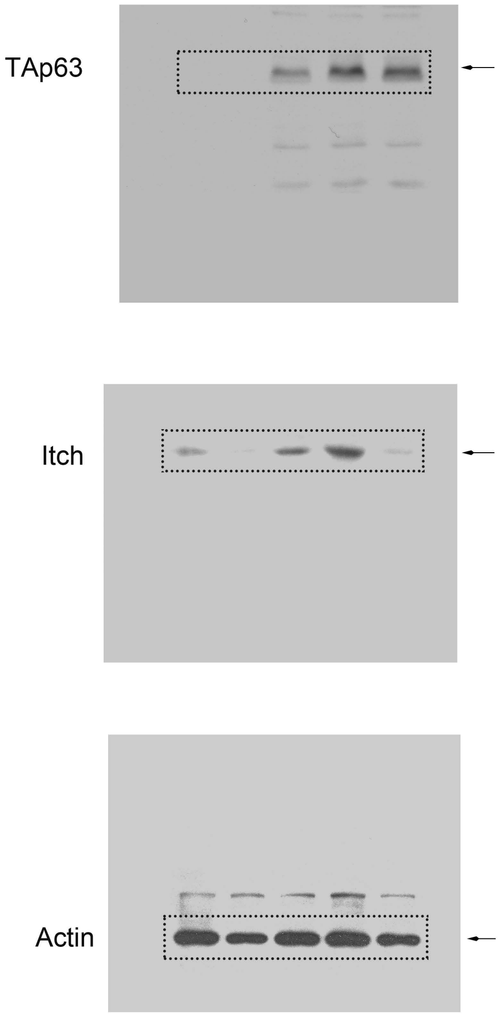

Supplementary Fig-1A

TAp63

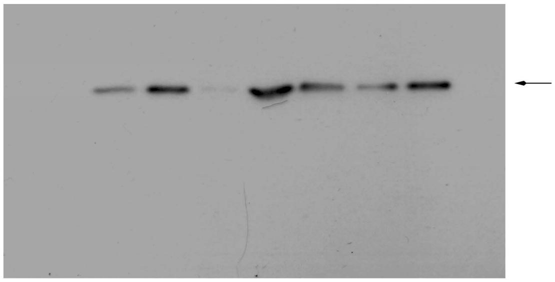

DNp63

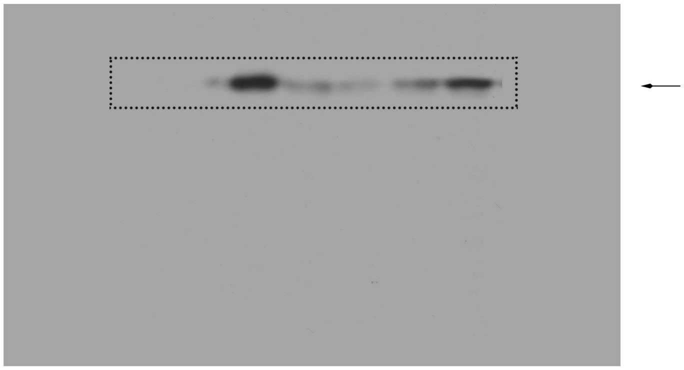

Ago2

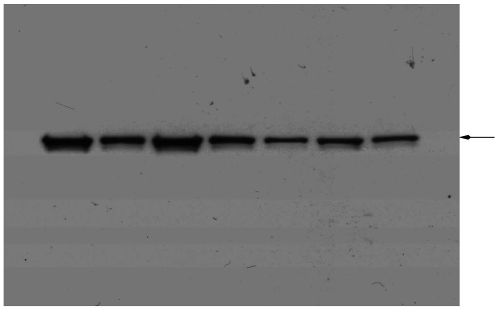

Actin

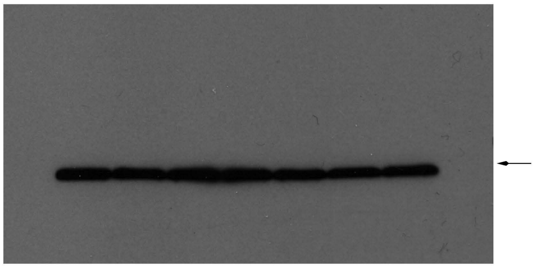

Suppl Fig-2A

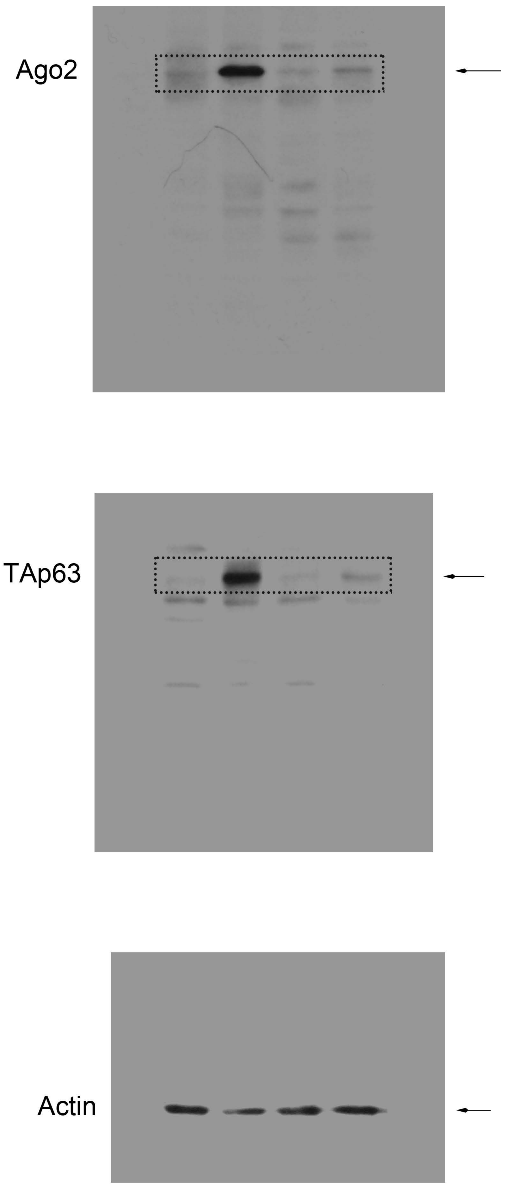

Suppl Fig-2B

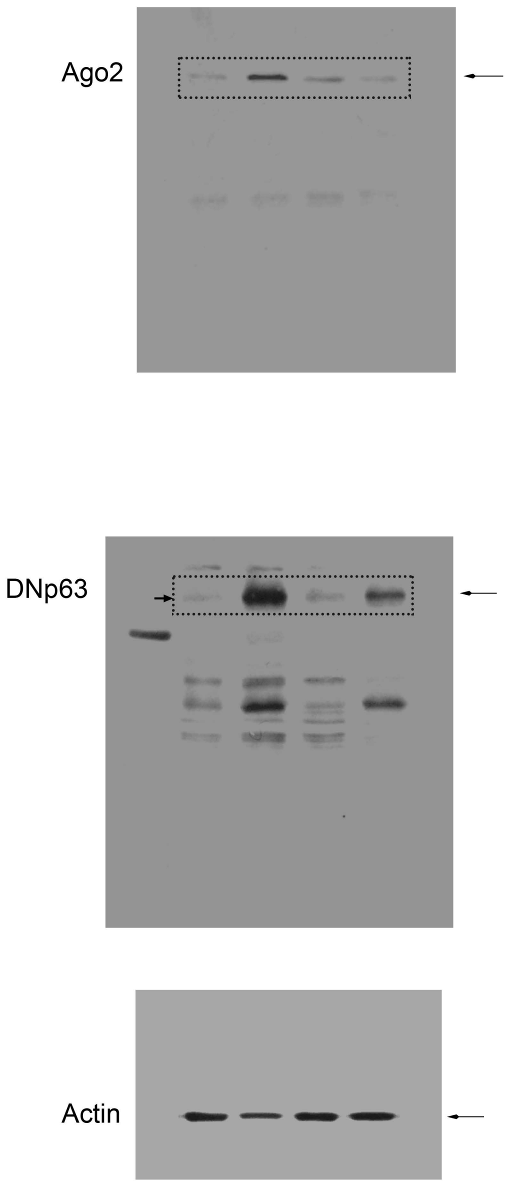

Suppl Fig 4E

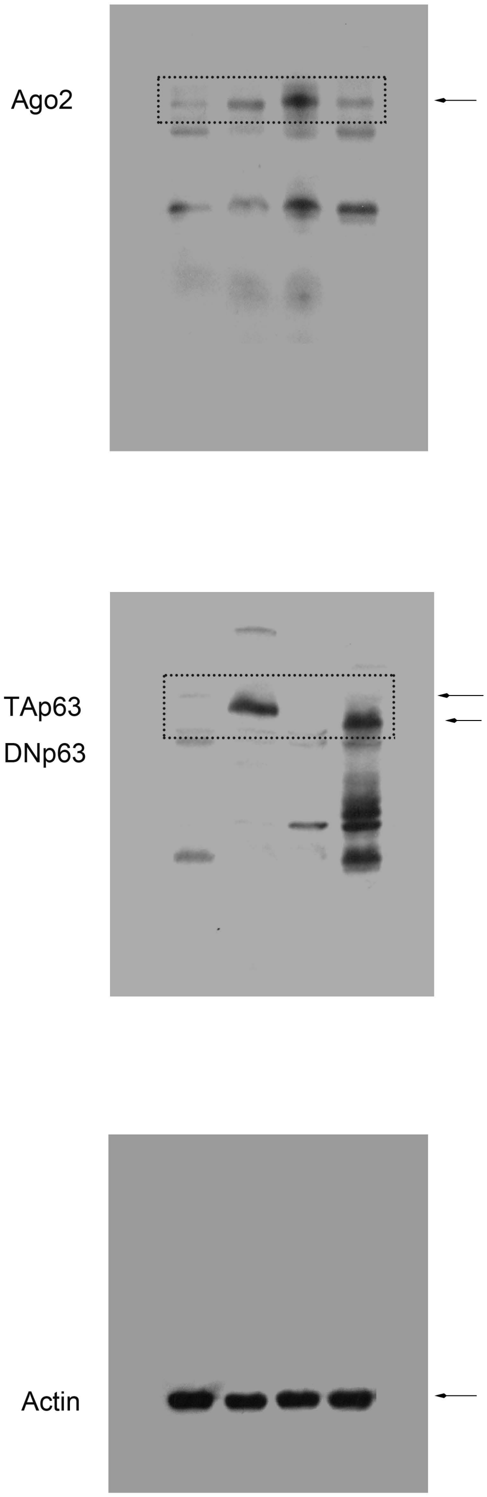

Suppl Fig 4F

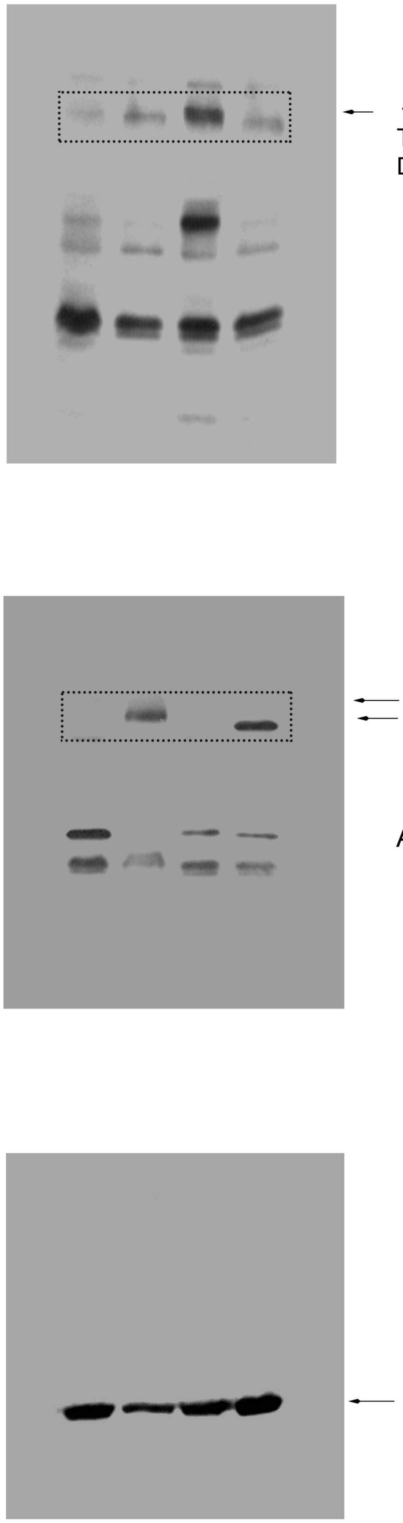

Suppl Fig 4H

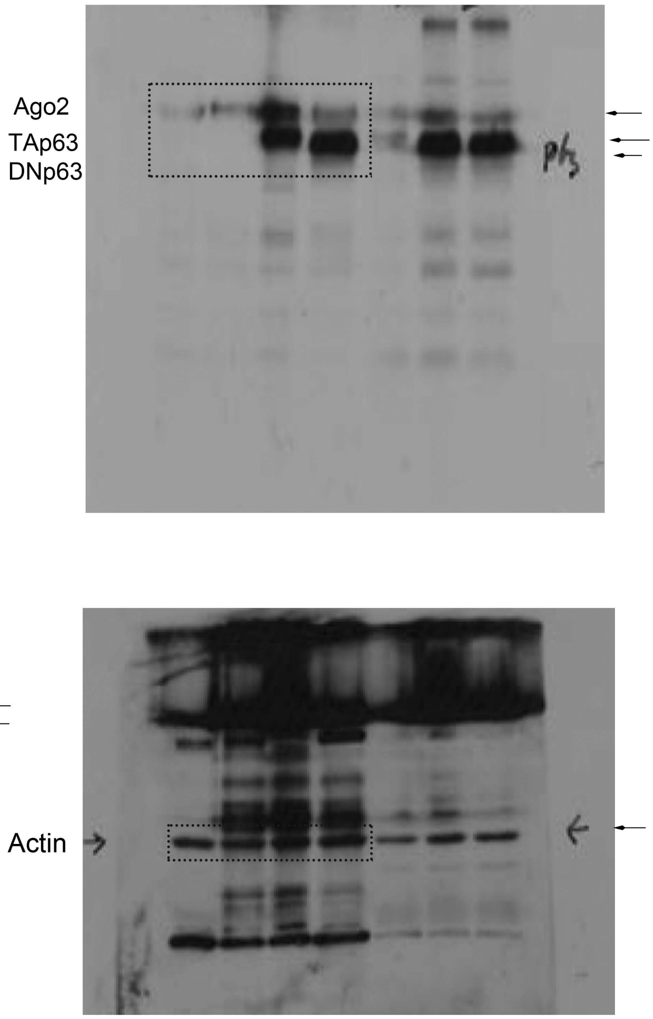

Suppl Fig-5B

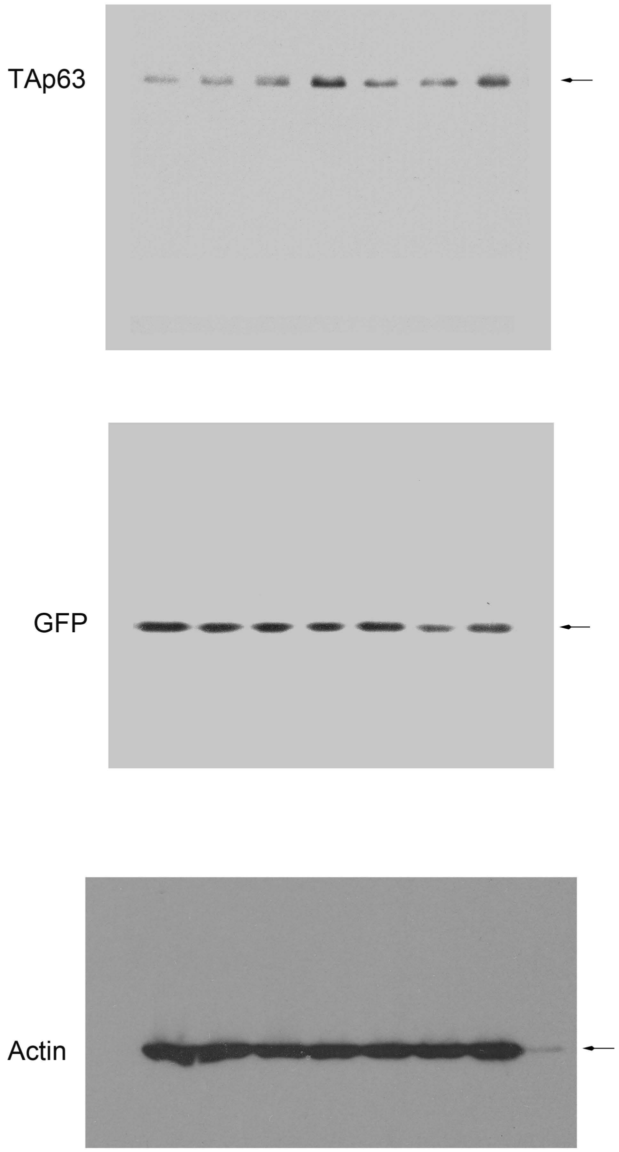

Suppl Fig-5C

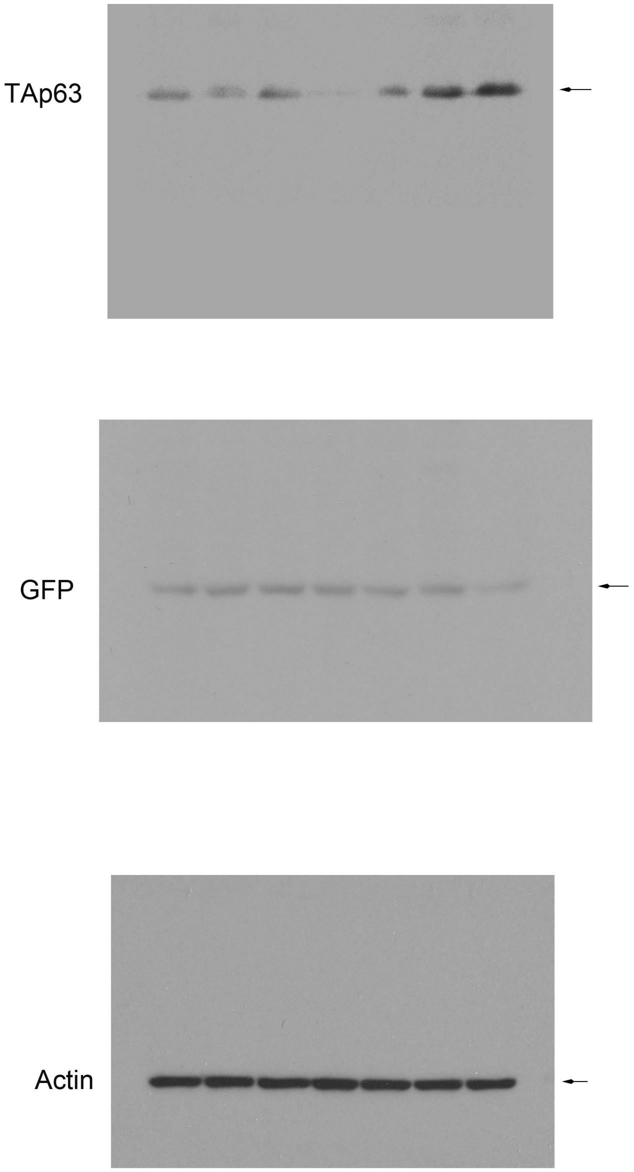

Suppl Fig 5D

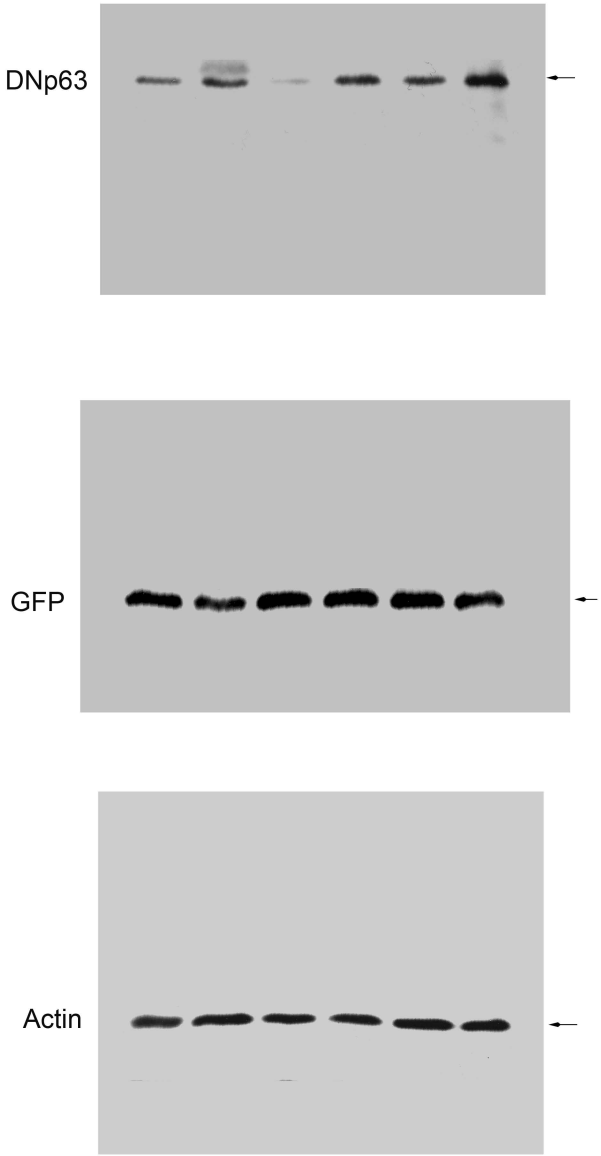

Suppl Fig 5E

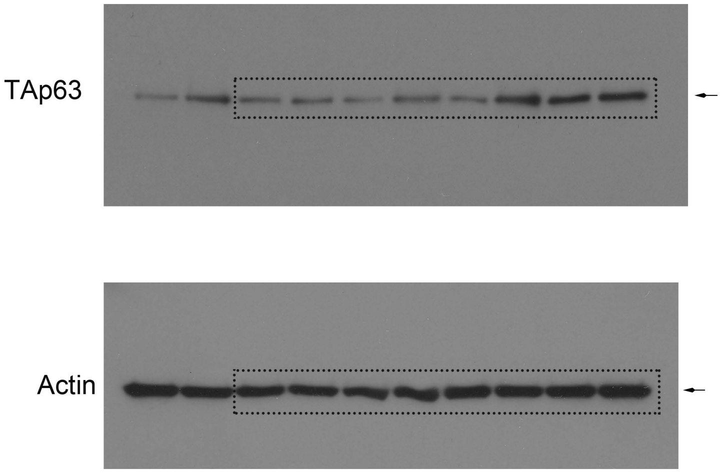

Suppl Fig 6A

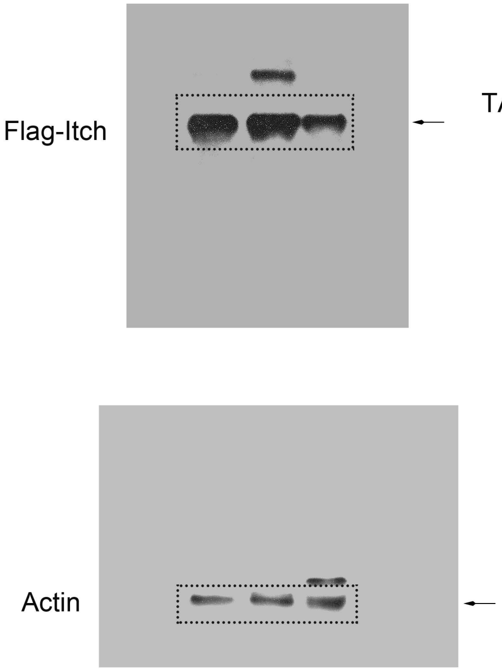

Suppl Fig 6F

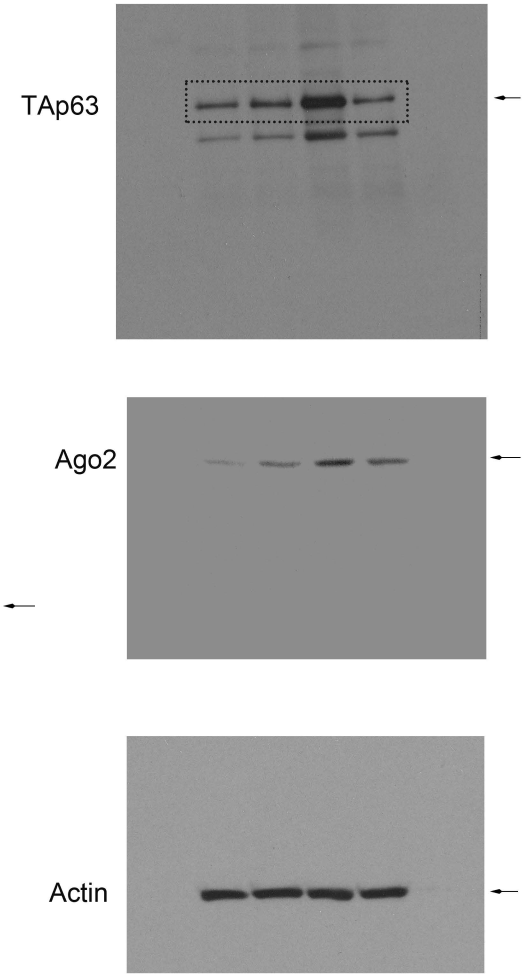

Supl Fig 6G

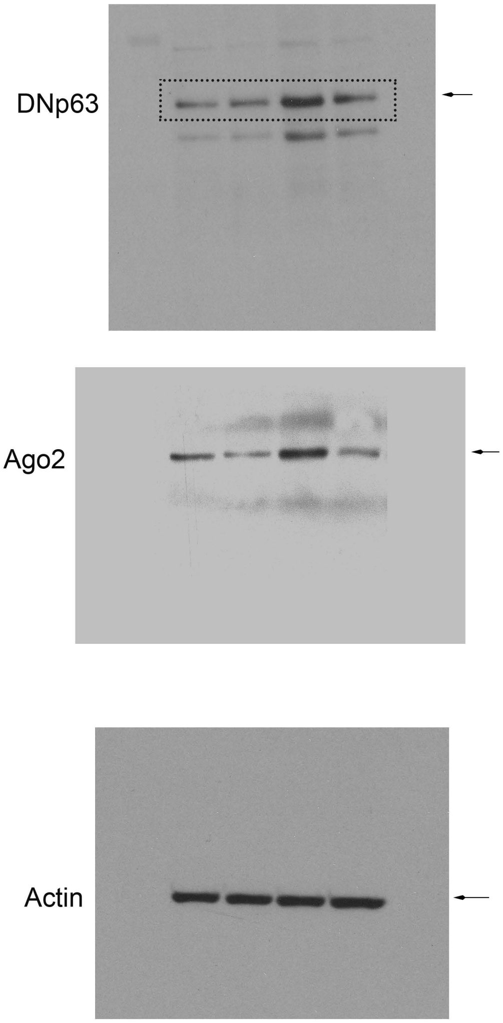

Supplement: Supplementary file 2 — Binder-original gels and blots for all figures [file 41419_2022_4854_MOESM2_ESM.pdf]
